# The dynamic mesoscale sink and source niches for eukaryotic phytoplankton in a subtropical gyre

**DOI:** 10.1073/pnas.2608700123

**Published:** 2026-06-17

**Authors:** Alexandra E. Jones-Kellett, Jesse C. McNichol, Yubin Raut, Jed A. Fuhrman, Michael J. Follows

**Affiliations:** ^a^ Departments of Earth, Atmospheric and Planetary Sciences, Massachusetts Institute of Technology, Cambridge, MA 02139; ^b^ Biology Department, Woods Hole Oceanographic Institution, Woods Hole, MA 02543; ^c^ Department of Oceanography, University of Hawai‘i at Mānoa, Honolulu, HI 96822; ^d^ Department of Biological Sciences, University of Southern California, Los Angeles, CA 90007; ^e^ Biology Department, St. Francis Xavier University, Antigonish, NS B2G 2W5, Canada

**Keywords:** oceanography, amplicon sequencing, Lagrangian, coherent structure, dispersal

## Abstract

By tracking the water mass histories of genetic samples, we investigated the biophysical dynamics shaping eukaryotic phytoplankton populations in a nutrient-deplete subtropical gyre, where cyanobacteria have a competitive advantage. Triplicate seawater samples were filtered every ∼46 km along a 2,382 km North Pacific transect, spiked with genomic internal standards, and amplified with a three-domain primer set to obtain absolute 16S and 18S rRNA volumetric gene abundances. The transport histories of each sample were simulated by advecting mesoscale Lagrangian particle clouds in satellite remote sensing velocity fields. Consistent with previous field studies, eukaryotic phytoplankton were anomalously abundant within eddies and along eddy-edges, where vertical circulations redistribute nutrients. Outside of eddies, we found a statistically significant decline in eukaryotes as a function of lateral coherence: Waters that recently mixed from multiple origins supported eukaryote anomalies resembling those of eddies, whereas eukaryotic populations were depressed from isolation in waters that were coherent for three or more months. In these coherent outside-eddy water masses, we estimate taxon-dependent eukaryote population half-lives range from 8 to 17 mo. Such physical conditions are relatively rare, given that >90% of the entire gyre during the sampling campaign was composed of eddies and recently mixed waters. These results derived from empirical observations substantiate the theory that eukaryotic phytoplankton would face exclusion in the subtropics on timescales of ∼years due to competitive pressure from cyanobacteria, yet are sustained in small numbers by regular disturbances promoting opportunistic growth and dispersal.

## Significance

Competition theory predicts that organisms with the highest evolutionary fitness for a particular environment will outcompete less fit species. Subtropical gyres are desert-like with low average nutrient concentrations, yet a diversity of phytoplankton types with various nutrient requirements coexist. Rotating vortices called eddies support this biodiversity by tapping into deep nutrient reserves via vertical mixing and boosting abundances of eukaryotic phytoplankton that may otherwise not survive in consistently low-nutrient conditions. Outside of eddies, we contrast two regimes: water masses with minimal lateral mixing for months penalize eukaryotes, whereas recently mixed waters contain eukaryotic population sizes reflecting those of mesoscale eddies. The results suggest a combination of eddies and horizontal mixing sustains microbial diversity in subtropical gyres.

**Fig. 1.**
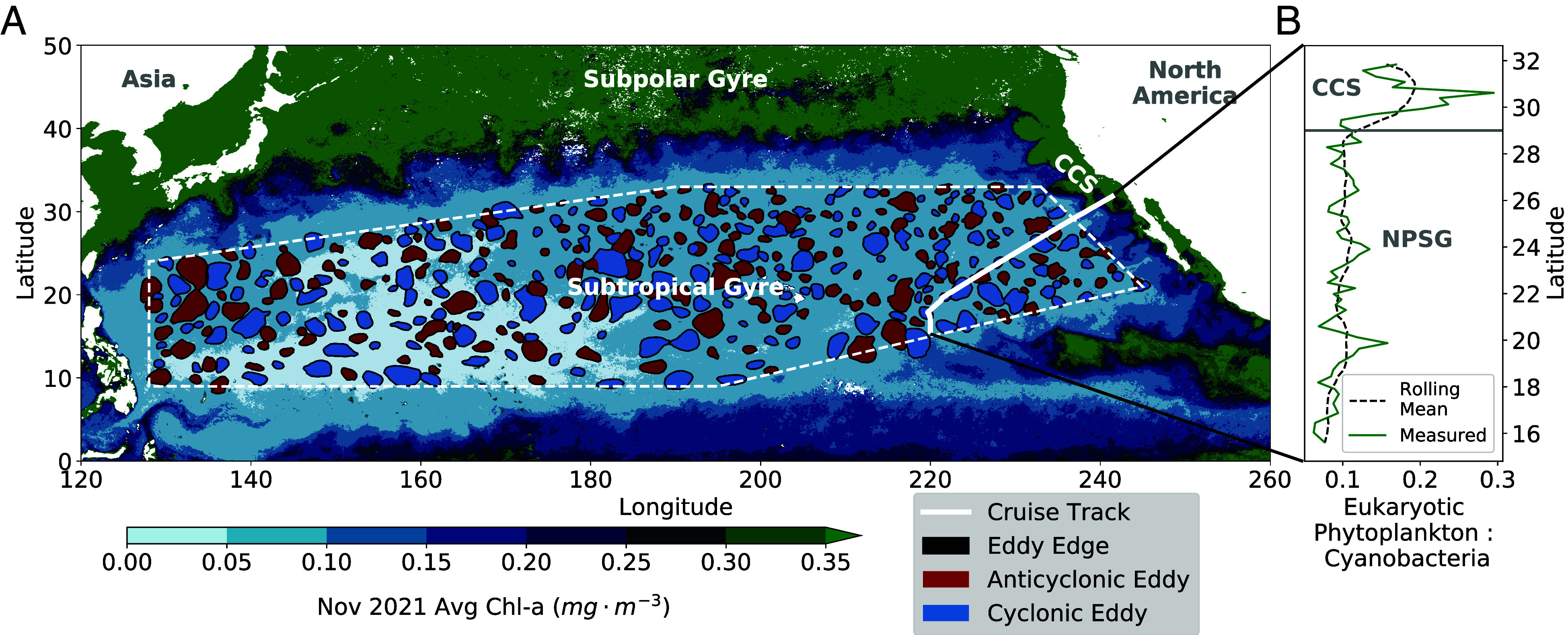
Regimes of phytoplankton biomass and community structure in the North Pacific. (*A*) The background shows the satellite surface chlorophyll-*a* average concentration (a proxy for phytoplankton biomass) in November 2021 (2). The subpolar gyre and California Current System (CCS) enhance phytoplankton concentrations, whereas biomass is low in the North Pacific Subtropical Gyre (NPSG). Mesoscale eddies located within the subtropical gyre polygon (white dashed line) on November 23, 2021, are superimposed on the map, revealing the ubiquity of these transient features (3). The solid white line represents the study cruise track. (*B*) The measured ratio of eukaryotic phytoplankton to cyanobacteria volumetric gene abundance is in green. The dashed line is the latitudinal rolling mean, revealing the large-scale biogeography: The ratio is higher in the coastal upwelling system and low in the oligotrophic gyre.

Marine phytoplankton fix CO_2_ into organic molecules that fuel food webs and maintain climatically important ocean carbon stores (1). In subtropical gyres, weak seasonality and climatological downwelling result in low nutrient supply rates and depressed phytoplankton biomass. In contrast, upwelling regimes and seasonal subpolar gyres provide regular nutrient enrichment that supports higher concentrations of phytoplankton (Fig. 1*A*). Furthermore, the community compositions of high and low biomass environments are distinct (Fig. 1*B*). The smallest cyanobacteria, *Prochlorococcus*, are most abundant in subtropical gyres (4) because, according to ecophysiological theory, their low subsistence nutrient concentrations provide a competitive advantage (termed “oligotrophs”) (5). In contrast, eukaryotes with higher maximum growth rates and larger cell sizes are fitter in nutrient-enriched environments (termed “copiotrophs”) (6, 7); there, they bloom transiently, and top–down forces foster coexistence (8–10). This paradigm of oligotrophic and copiotrophic phytoplankton provides a conceptual model of basin-scale biogeography that underpins most modern marine ecosystem and climate models (11).

Despite competitive pressure from oligotrophic cyanobacteria, small numbers of copiotrophic eukaryotic phytoplankton are still ubiquitous in the ocean’s subtropical gyres. Local increases in eukaryotes within the North Pacific Subtropical Gyre (NPSG) have been attributed to mesoscale (O(10 to 100 km)) eddies, which generate vertical motions that can temporarily enrich the sunlit layer with nutrients from below (12–17). However, only half of NPSG eddies laterally trap waters for as long as a month (18), while “leaky” eddies rapidly transport new blooms across eddy boundaries into surrounding waters (19). This suggests that dispersal may play a key role in propagating eddy-affected populations to new locations (20–23). Here we test the hypothesis that without the combination of eddy disturbances and subsequent lateral mixing, a steady pressure of competitive exclusion from *Prochlorococcus* will act to deplete eukaryotic phytoplankton populations in the NPSG. To do so, we use in situ field amplicon sequence data coupled with Lagrangian flow analysis to observe the mesoscale source and sink regions for eukaryotes.

We conducted a shipboard sampling campaign, collecting surface seawater in triplicate at a high-density spatial separation of 46 km for a distance of 2,382 km. This equidistant sampling strategy provided a comprehensive survey of waters within, near, and outside of mesoscale eddies. We amplified 16S and 18S rRNA genes using a universal primer set, simultaneously characterizing prokaryotic and eukaryotic Amplicon Sequence Variants (ASVs) (24, 25). Additionally, we spiked each sample with genomic standards, yielding quantitative gene abundances previously validated with concurrent flow cytometry measurements (26). We evaluated the volumetric gene copies as a function of mesoscale eddy presence and water mass transport history, derived from Lagrangian particle simulations in satellite altimetry observations. Our approach links observations of eukaryotic phytoplankton abundance in the NPSG to mesoscale forcings, including lateral isolation, dispersal, and eddies.

## Results

### Phytoplankton Spatiotemporal Anomalies.

**Fig. 2.**
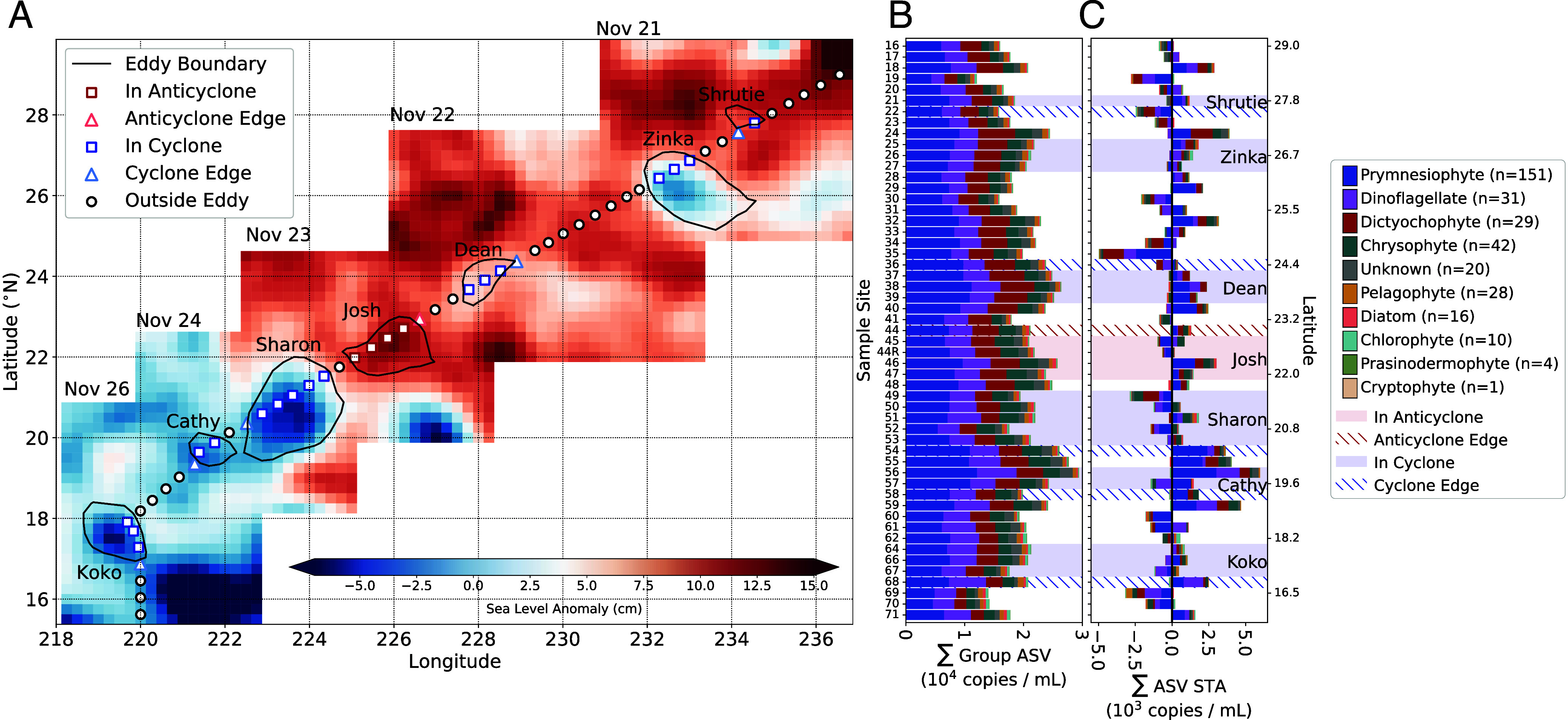
NPSG amplicon sample collection locations, eukaryote group abundances, and anomalies. (*A*) Map of the sample locations, plotted as squares if in a mesoscale eddy, triangles if on an eddy edge, and circles if outside eddies. Eddies are referred to by the labeled names. Daily sea level anomaly (SLA) “tiles” in the background (29) are in alignment with the dates the samples were collected. (*B*) Stacked group-level sums of eukaryotic phytoplankton abundances and (*C*) spatiotemporal anomalies (STAs). The blue and red background shading delineates cyclonic and anticyclonic eddy regions, respectively. The hashed samples are eddy edge. The legend includes the number of ASVs in each taxonomic group (n=x). *SI Appendix*, Fig. S3 is an equivalent plot for cyanobacteria.

The data include 53 triplicate NPSG surface samples collected over a week along a transect spanning 13.4 latitudinal degrees (*SI Appendix*, Table S1). Trends in volumetric 16S and 18S gene abundance at the domain, mixed taxonomic group (e.g., class or phylum), and individual ASV levels along the subtropical transect evidence gyre-scale biogeographic variability (*SI Appendix*, Fig. S1). This may be attributed, for example, to thermal dependencies (27); the sea surface temperature (SST) ranged from 19.1 to 25.7 °C, increasing linearly from north to south. Perturbations in gene abundance from mesoscale processes are of higher frequency, but lower magnitude than the gyre-scale biogeography (e.g., see the measured abundances versus the rolling mean in Fig. 1*B*). To isolate mesoscale-driven biological reactions, we computed a latitudinal anomaly based on deviations in gene abundance from a rolling mean (*SI Appendix*, Fig. S2 *A* and *B*). The latitudinal anomaly subsequently revealed diel fluctuations in gene abundance (*SI Appendix*, Fig. S2*C*), attributable to daily cycles of light availability, cellular division rates, grazing pressure, and viral lysis (28). These diel rhythms are present irrespective of mesoscale processes, so we also removed the temporal trends in gene abundance, yielding a spatiotemporal anomaly (STA) for each ASV (Fig. 2*C*; methodology detailed in *SI Appendix*, *Spatiotemporal Anomaly*). We note that the diel cycles have only a modest effect on the STAs because the latitudinal variations in gene abundance are of much greater amplitude.

The STA quantifies whether the abundance of each ASV is enhanced or depressed, given the location and time since sunrise. We interpret these anomalies as potential responses to local mesoscale flows and perturbations. Notably, the anomaly computation was enabled by an internal standard correction that allowed us to backcalculate PCR amplification factors. Without standards, amplification factors are unknown, and quantitative changes in gene abundance are not easily interpretable between samples (30). Although rRNA gene copy numbers per cell vary for eukaryotes, previous work has shown that quantitative metagenomic-derived abundances of eukaryotic phytoplankton groups scale with flow cytometry cell counts (31) and carbon biomass (32). Therefore, we assume that the quantitative volumetric gene abundances scale with population sizes for this analysis.

### Mesoscale Water Mass Properties.

We hypothesized that the microbial community observed at any given time and place reflects the mesoscale advective history in recent months and environmental disturbances experienced by the host water mass. To test this, we first categorized each biological sample as collected “in eddy” (N=21), on an “eddy edge” (N=6), or “outside eddy” (N=26; Fig. 2*A*). Samples were collected in six distinct cyclonic mesoscale eddies (counterclockwise rotation) and one anticyclonic (clockwise), identified from satellite altimetry (3). The eddy lifespans and ages varied and are reported in *SI Appendix*, Table S2. Eddy edge samples include those collected within 15 km of the eddy boundary. Distinguishing these samples from other outside-eddy samples accounts for the possible error in eddy boundary detection (33), perturbations along eddy peripheries due to vertical velocities induced by ageostrophic submesoscale flows (34, 35), and lateral exchange between eddies and outside waters (19).

Next, to capture the advective histories of the sampled waters, we simulated Lagrangian trajectories in satellite-derived velocity fields. We initialized Gaussian distributed clouds of Lagrangian particles around each sample site with a spread of $0.025^{{}^{\circ}}$, roughly 20 km in diameter. The clouds have the highest density at the sampling location. We repeated this experiment in two velocity products: one that assumes the geostrophic approximation (29) and another that additionally incorporates an Ekman component (36). When advection was derived from the geostrophic flow, we found that anomalies of SST correlate with the extent of meridional excursions along the background gradient for time periods of 3 to 6 mo, consistent with a mixing model. This was not the case when the Ekman flow was included (*SI Appendix*, Fig. S5). Therefore, we infer that the derived geostrophic advective pathways best represent mesoscale heat and tracer transport in our domain (37), and thus assess the biological response to geostrophic currents (see *SI Appendix*, *Justification of Geostrophic Approximation* for more detail).

**Fig. 3.**
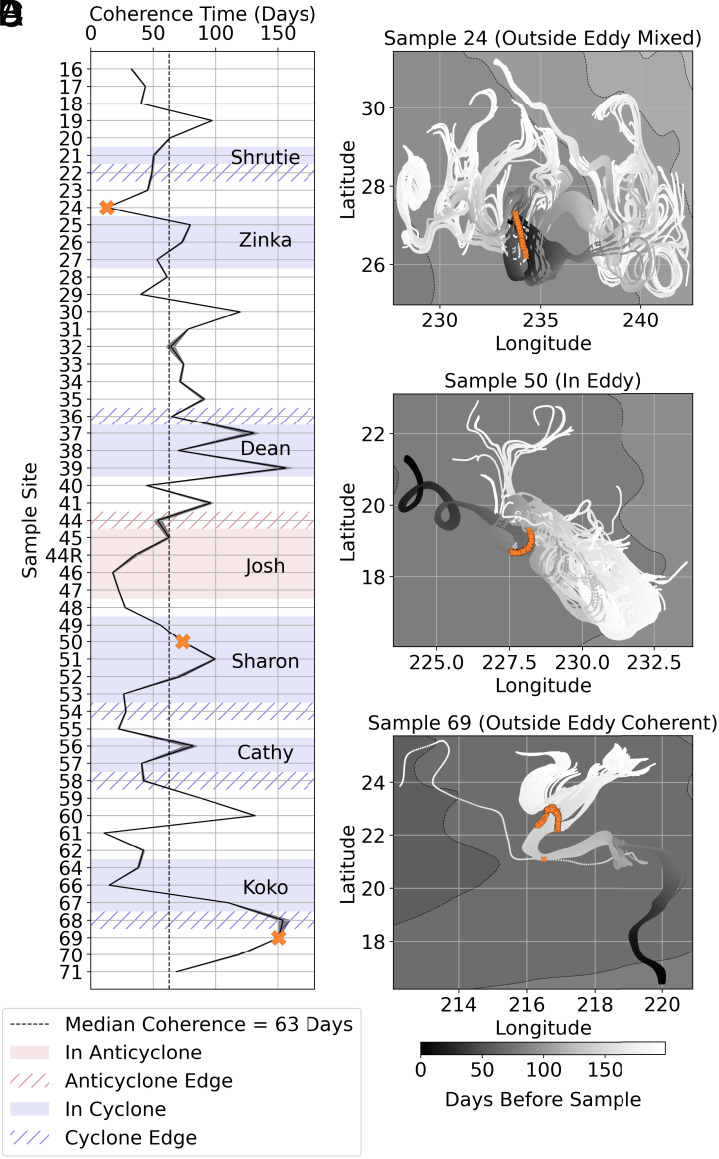
Lagrangian coherence times with select trajectory visualizations. All trajectories are shown in Movie S1. (*A*) The Lagrangian coherence time for the water masses associated with each biological sample. Low coherence time indicates a recent history of lateral mixing, whereas a high coherence time suggests minimal lateral exchange. The gray shading represents the range of coherence times measured from three particle cloud initializations per sample. The eddy shading is synonymous with Fig. 2 *B* and *C*. (*B*–*D*) Example Lagrangian trajectories of the Gaussian clouds initialized on the sample sites, which were used to derive the coherence time. The particle locations at the time for which coherence was lost are marked with orange X symbols that correspond to the X’s in panel (*A*).

The geostrophic trajectories of the Gaussian clouds are shown in Movie S1, tracing the origins of the water parcels before they converged upon the sample sites. The variability in lateral mixing behaviors is striking: Some clouds traveled as isolated entities for several months (e.g., Fig. 3*D*), while others were intensely mixed, with waters originating from widespread directions (e.g., Fig. 3*B*). To quantify the extent of lateral mixing, we define the geostrophic coherence time of each cloud as the point at which the mean particle displacement is twice the initialization distance from the center of mass. The coherence time among the sample sites ranges from 10.6 to 156.2 d and varies along the transect without apparent latitudinal dependence (Fig. 3*A*). Note that this definition of water mass coherence time is distinct from the eddy retention time (i.e., the time particles spend retained inside an eddy boundary). The coherence time of samples collected inside mesoscale eddies is 65 d on average, similar to the mean of 67 d for samples collected outside eddies.

### Phytoplankton Signature of Coherence Time.

**Fig. 4.**
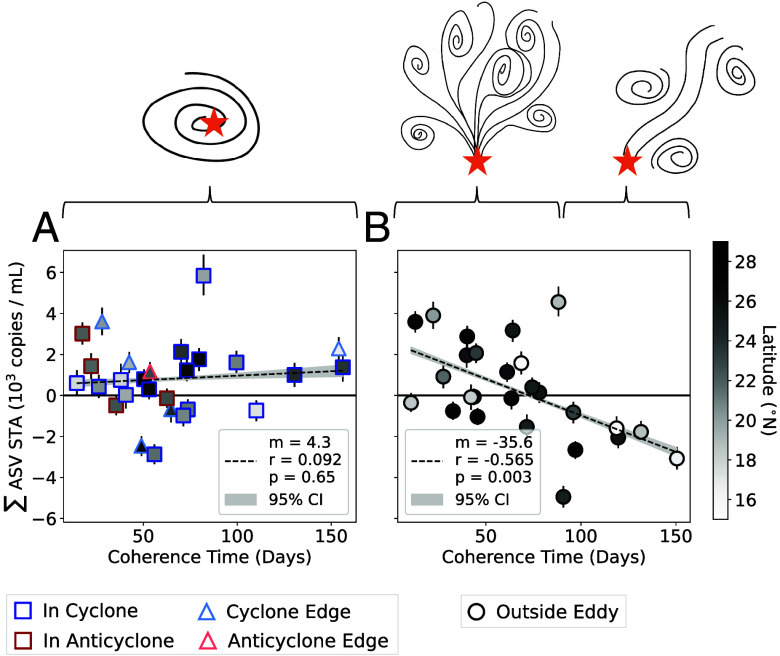
Sample sums of eukaryotic phytoplankton ASV STAs as a function of coherence time. (*A*) The sum of eukaryotic phytoplankton STAs in eddy-associated samples, with blue outlines corresponding to cyclones, and red outlines to the anticyclone. The triangle shape indicates an eddy edge sample, while the squares indicate in-eddy. The interiors of the scatter points are colored by latitude. The legend includes the slope of the linear fit (m), the Pearson correlation coefficient (r), and the *P*-value (p). The gray shaded region shows the 95% CI (CI) for the regression. The vertical black lines on each scatter point show the 95% CIs for the STA values. (*B*) Same as (*A*), but for outside-eddy samples. Here, the regression is statistically significant (P=0.003). The schematics above the plot provide a visualization of the trajectory histories based on coherence time: Waters with low coherence time have different origins that converged upon the sample site (represented by the star), whereas long coherence time indicates extended isolation from the surroundings.

With quantitative metrics for both mesoscale biological and physical perturbations, we tested whether sample sites with highly dispersive advective histories supported anomalously greater eukaryotic phytoplankton populations than laterally coherent waters. At the domain level, we found no statistically significant relationship between cyanobacteria and coherence time (*SI Appendix*, Fig. S8). The eukaryotic phytoplankton tell a different story: STAs are indeed significantly linearly dependent on coherence time (Pearson’s r=−0.565, *P*-value = 0.003), decreasing in abundance with isolation time for samples collected outside of eddies (Fig. 4*B*). This trend is maintained for the most abundant eukaryotic phytoplankton taxonomic groups, including Prymnesiophytes, Dinoflagellates, Dictyochophytes, Chrysophytes, and Pelagophytes (*SI Appendix*, Fig. S9*A*). The rarer taxonomic eukaryote groups (Diatoms, Chlorophytes, Prasinodermophytes, Cryptophytes, and Unknown), which contributed as a whole <13.1% of the eukaryotic phytoplankton genes analyzed in each sample, do not exhibit a significant relationship with coherence time (*SI Appendix*, Fig. S9*B*).

Given the observed relationship outside of eddies, we conducted a thought experiment modeling the average rates of population decline for the most abundant eukaryotic phytoplankton populations in a laterally coherent water mass, if coherence were to be maintained indefinitely. While the data in Fig. 4*B* are compatible with a linear decline, over longer timescales, we would expect population sizes (C, gene copies/mL) to decay exponentially, such that

**Fig. 5.**
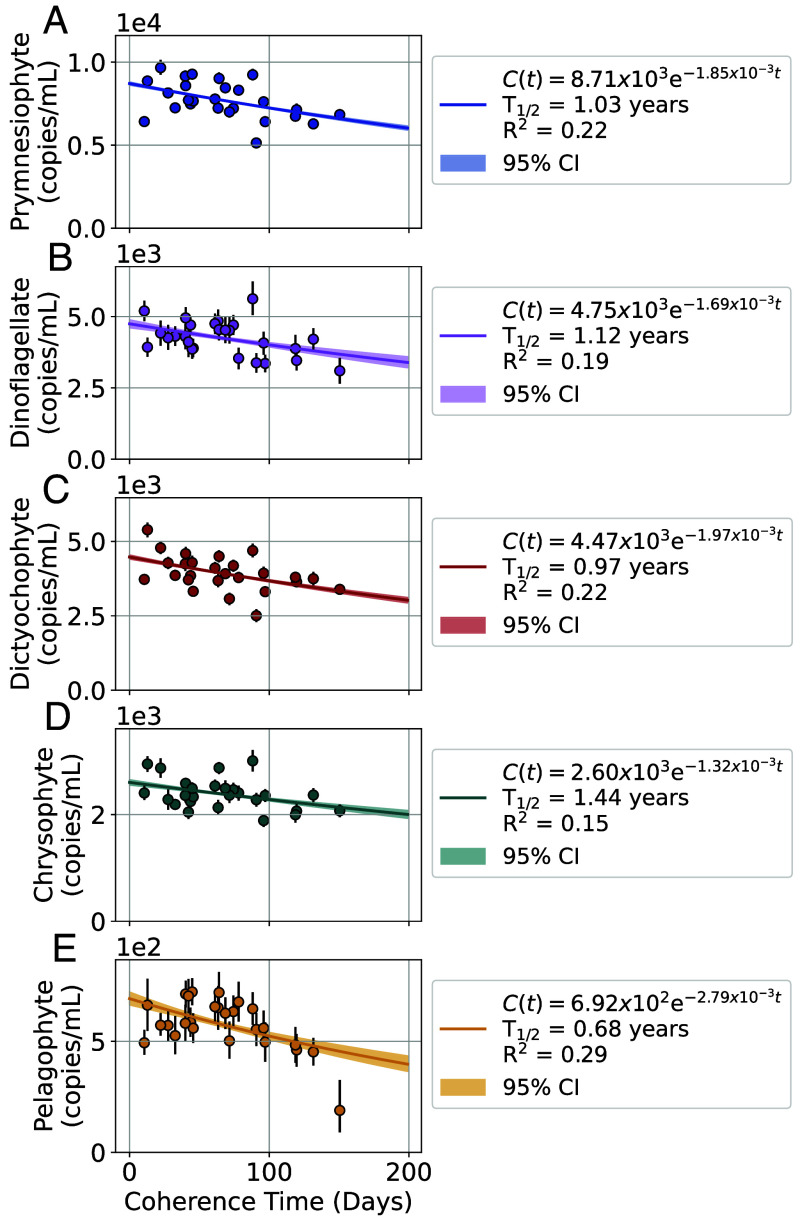
The exponential decay of abundance in outside-eddy samples as a function of coherence time for (*A*) Prymnesiophytes, (*B*) Dinoflagellates, (*C*) Dictyochophytes, (*D*) Chrysophytes, and (*E*) Pelagophytes. Note that the y-axis differs for each subplot. The scatter plot data were generated by adding the outside-eddy STAs to the group’s average volumetric abundance across all samples, representing the average expected evolution of the population abundance with time in a coherent water mass in the sampled domain. The vertical black lines on each scatter point show the 95% CIs for the estimated abundance. Note that the linear fits of these data (*SI Appendix*, Fig. S9*A*) are nearly identical to the exponential fits for the timescale of 200 d shown here. The equations for the exponential fits (C(t)) are in units of days, while the half-lives ($T_{1/2}$) are reported as units of years. The shaded regions show the 95% CIs for the exponential fits of the data.

[1] $$\begin{array}{c} C\left(t\right)=C_{0}e^{\mu t}, \end{array}$$

where $C_{0}$ is the initial population size, μ is the net negative growth rate, and the population half-life is $T_{1/2}=\frac{ln(2)}{\mu }$ (see mathematical derivation in *SI Appendix*, *Tracer Equation Scaling*). To extrapolate the rate of decay of eukaryotes in an indefinitely coherent water mass, we transformed the STA data to synthetic abundances by adding the average abundance of each eukaryote group (data provided in *SI Appendix*, Table S3) over all samples to the outside-eddy STA data. The transformed data represent the average expected abundance of a population in this region as a function of the observed coherence times, while remaining independent of latitude and time of day. We then fit the exponential decay model to derive the half-lives of each eukaryote group. The shortest half-lives of the eukaryote groups is $T_{1/2}=0.68$ y for Pelagophytes, and the longest is 1.44 y for Chrysophytes (Fig. 5). Thus, we estimate that the volumetric abundance of all major eukaryotic populations would decline significantly over the timescale of a year or so if remaining in an isolated, noneddy regime. However, like mesoscale eddies, these Lagrangian coherent structures are transient.

### Phytoplankton Signature of Mesoscale Eddies.

Independent of a sample’s water mass coherence time, eukaryotic phytoplankton abundances are, on average, anomalously positive in eddy-associated samples (*SI Appendix*, Table S3). This is the case for the whole eukaryote domain and for each of the most abundant groups (i.e., those in Fig. 5), all with positive 95% CIs. Eukaryote enhancement in eddies of both polarities is consistent with the benefits of enriched nutrients via eddy pumping in the center of cyclonic eddies (38, 39), by wind-driven mixing in anticyclones of subtropical gyres (40, 41), and vertical velocities induced along eddy edges (34, 35). Thus, vertical processes are likely the primary influence on elevated eukaryote populations in eddies, as opposed to the lateral history of the water mass. We note that it is unclear how broadly representative the anticyclone observations are, however, since we only sampled one of these features.

Despite the average eukaryote enhancement in eddies, the magnitudes of the anomalies have high variability (see SDs in *SI Appendix*, Table S3), and we did not find a common community response to the observed eddies; each stimulated various taxa to different extents (Fig. 2*B*). Prymnesiophytes have the most positive STA summed across the samples collected from Cyclones Dean, Cathy, and Shrutie. Chrysophytes were the most anomalously abundant within Cyclone Sharon and Anticyclone Josh. Dictyochophytes were the biggest contributor to the STA in Cyclone Koko, while dinoflagellates were the group most enhanced in Cyclone Zinka. We found anomalously positive eukaryotic phytoplankton gene abundance cumulatively within the lone anticyclone for all groups except for Pelagophytes and Prasinodermophytes. Sample 46 of Anticyclone Josh had the second-highest diatom STA, after Sample 51 in Cyclone Sharon. Overall, we found that eddies of either polarity can perturb the eukaryotic phytoplankton community, but there is high variability in the response to any given eddy.

**Fig. 6.**
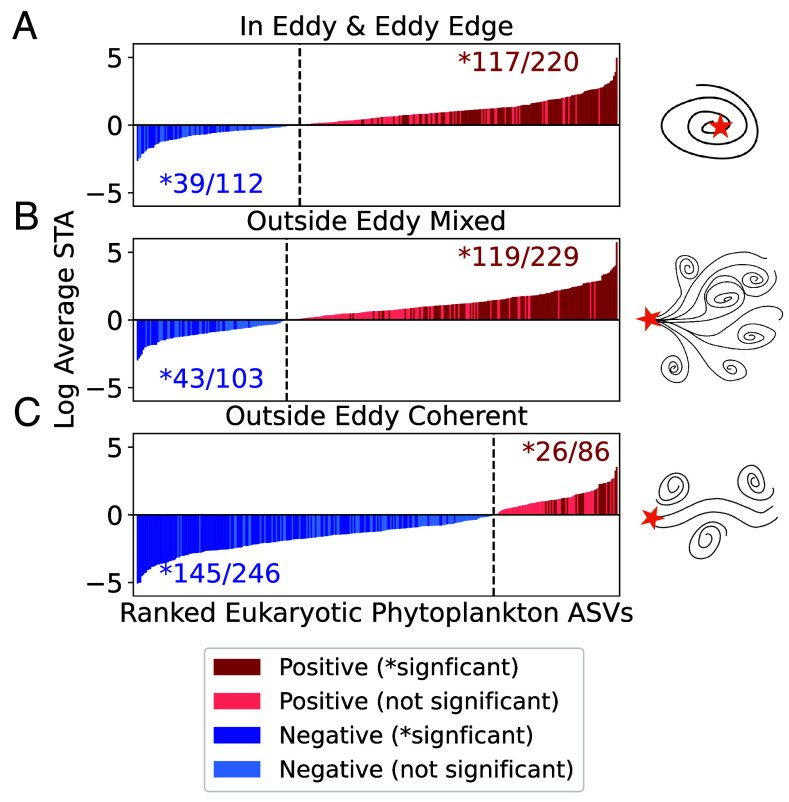
Ranked average eukaryotic phytoplankton STAs across samples of each mesoscale niche. (*A*) Ranked average ASV STAs from eddy and eddy edge samples. The data are log-modulus transformed for visual purposes (i.e., sign(STA)×ln(|STA|+1)), accommodating negative and positive values. ASVs with positive anomalies on average are colored red, and negative blue. The darker colors represent ASVs that have a 95% CI of the same sign, i.e., significantly positive or negative. The total fraction that is significant is denoted by the ∗. A nonsignificant sign of anomaly (light colors) is primarily due to high variability or low abundance among samples. The black dashed line delineates a change in sign from negative to positive. The data for each ASV are provided in *SI Appendix*, Table S4. (*B*) Same as (*A*) but for outside-eddy samples recently mixed within the past 90 d. (*C*) Same as (*A* and *B*) but for outside-eddy samples that were coherent for 90 or more days.

We compared phytoplankton anomalies in eddies with those outside to test if eddies uniquely stimulated local phytoplankton communities. We divided the outside-eddy samples into two comparison groups: waters that were coherent for 90 or more days (i.e., the 25% most coherent) and those that mixed waters with distinct origins within 90 d. As in eddies, the average eukaryote STA in recently mixed waters is anomalously positive for all abundant eukaryote groups (with positive 95% CIs, *SI Appendix*, Table S3). On the other hand, outside-eddy coherent waters have anomalously depressed populations for these same groups (with negative 95% CIs, *SI Appendix*, Table S3). Examining the average STA in each water mass type for the 332 individual eukaryotic phytoplankton ASVs in the analysis, a similar number of ASVs were anomalously positive in the eddy samples (220; Fig. 6*A*) to that of the outside mixed (229; Fig. 6*B*), with a shared 119 ASVs that were anomalously positive in both. In contrast, 246 of the ASV STAs were negative on average in the outside-eddy coherent waters (Fig. 6*C*). These results suggest similar phytoplankton signatures of eddies and recently mixed waters with divergent trends in the coherent outside-eddy water masses.

## Discussion

Our data resolve microbial community composition at the mesoscale over a large swath of a subtropical gyre, allowing us to examine the relationship between phytoplankton gene abundance and local horizontal flows. We consider these relationships in the context of the underlying, idealized paradigm that the smallest cyanobacteria have a competitive advantage for limiting resources under relatively quiescent oligotrophic conditions, driving nutrient concentrations below the subsistence resource concentration for larger eukaryotes (5). However, disturbances and transient nutrient enrichments favor blooms of faster-growing, opportunistic eukaryotes. We distinguish between three distinct mesoscale regimes in terms of eukaryotic phytoplankton abundance anomalies (Fig. 4): i) Water masses inside or on the edge of eddies, likely associated with ongoing or recent vertical disturbances in the resource environment. This regime is characterized by the enhancement of eukaryotic phytoplankton, potentially mediated by eddy pumping (38, 39), wind-interactions (40, 41), and/or submesoscale flows (34, 35). ii) Waters outside of eddies, but influenced by nearby eddies and/or recent disturbances through intense lateral mixing with neighboring waters, providing immigrant sources of eukaryotes (21). iii) Outside-eddy water masses that have remained laterally coherent for three or more months are characterized by a depletion of eukaryotic phytoplankton due to a systematic decline in abundance with isolation time. This reflects a dynamic adjustment of the ecosystem toward a more oligotrophic system, in which every major taxonomic eukaryotic phytoplankton group would halve in size in 8 to 17 mo if the structure were to be maintained indefinitely (Fig. 5).

Surface eukaryotic phytoplankton population sizes were enhanced on average within mesoscale eddies and along their peripheries, consistent with the theory that these physical flows fuel primary productivity in subtropical gyres (42). Given that more than a third of NPSG waters were in an eddy or eddy edge during the sampling campaign (see eddy coverage in Fig. 1 and *SI Appendix*, Fig. S11*D*), by extension, it is expected that they contribute substantially to the maintenance of opportunist phytoplankton across the gyre. Nevertheless, the anomaly magnitudes and the benefited taxonomic groups were inconsistent among the multiple sampled eddies as well as within any given eddy. Since each eddy was sampled within the same week and embedded in a similar gyre environment, the results suggest that it may not be appropriate to make generalizations about the specific taxonomic response to eddies by extrapolating from single-eddy observations. Additionally, the results imply that eddies act to increase diversity more so than if each feature exhibited the same taxonomic response. A recent study likewise revealed highly variable bacterial community compositions among 26 Indian Ocean eddies (43); they found that age, amplitude, and speed were predictors of diversity, although these relationships were weak. The eddies observed here had vast age differences, ranging from 1 (Cyclone Shrutie) to 326 d old (Cyclone Sharon) at the time of sampling (*SI Appendix*, Table S2), which may have contributed to biological variability (40). Diversity in grazing pressures and stochastic variations in the seed populations present during the eddy spin-ups could be added factors determining the ecological responses to disturbance.

We measured the Lagrangian trapping strength of each sampled eddy to test if the underlying physics may have driven biological differences in these features. Notably, no samples were collected inside a Lagrangian coherent vortex (*SI Appendix*, Fig. S10). This suggests that the in-eddy waters experienced considerable exchange with their surroundings, potentially laterally diluting the local phytoplankton response to physical disturbance (19). Furthermore, given the prevalence of eddy “leakiness” in the NPSG (18), eddy-driven disturbances are viable sources of eukaryotes into the background gyre via dispersal. This is supported by similar numbers of enhanced eukaryote ASVs in eddy-associated waters and outside-eddy mixed waters (Fig. 6), with a large fraction of the same ASVs enhanced in both niche types. We interpret the eukaryote enhancement in outside-eddy mixed waters as the biological memory of nearby perturbations, with immigrant populations being dispersed to new locations (20–23). For example, the spiraling swirls in the trajectories of Fig. 3*B* show the variety of transport pathways back in time to multiple eddy origins. Mixed waters may also carry the biological signature of fronts, another source of population-level perturbations (44) and heterogeneity (45).

Outside-eddy regimes that were laterally coherent for three or more months (e.g., Fig. 3*D*) provide an unfavorable environment for eukaryotic phytoplankton. Biological isolation has been explored in coherent vortices that generate nutrient-replenishing vertical circulations (34, 35, 38, 39), ultimately creating temporary ecological refuges that enhance competition (46, 47). Instead, the coherent waters outside of eddies reflect isolation in a progressively oligotrophic state, given the consistent decreases in abundance across eukaryote taxonomic groups (*SI Appendix*, Fig. S9*A*) and unique ASV-level community structures in these samples (Fig. 6). From a Lagrangian analysis across the entire NPSG, we estimate that at least 7% of mesoscale water masses during the sampling period were coherent for 90 or more days (*SI Appendix*, Fig. S11). Thus, >90% of the NPSG was actively being disturbed by eddies and dispersive currents that carry the imprint of recent disturbance, which sustain small populations of eukaryotic phytoplankton despite the fitness advantages of *Prochlorococcus* in the mean state. Coherence extending 200 d is rare in the NPSG (*SI Appendix*, Fig. S11*C*), such that mixing is likely to occur before eukaryote populations are substantially depleted, based on the observed rates of change with isolation time (Fig. 5).

While lateral coherence penalized eukaryotic phytoplankton during our study period (i.e., the summer and fall months), during the winter, atmospheric storms and surface heat loss lead to vertical mixing and convection that deliver nutrients to the euphotic zone. A wintertime peak in Turbulent Kinetic Energy (TKE) drives chlorophyll blooms across subtropical gyres (48) and has been observed to stimulate eukaryotic phytoplankton at Station ALOHA in the NPSG (49). Our survey occurred in November at the end of the seasonal quiescent period, so atmospheric disturbances had the smallest influence on the system during the prior months of focus in this analysis (*SI Appendix*, Fig. S12). A stormy winter may produce a quite different regime in coherent waters, but is left to future investigation. We additionally note that geostrophically coherent mesoscale water masses are subject to unresolved lateral submesoscale flows. If eukaryotic population sizes are relatively larger outside of a coherent water mass, submesoscale mixing across the boundaries may act to slow the rate of decline of eukaryotic phytoplankton populations (*SI Appendix*, *Tracer Equation Scaling*). While beyond the scope of this mesoscale observational study, submesoscale effects and the cascading associations between the mesoscale and submesoscale (50) could be further explored with a high-resolution numerical simulation.

Given that phytoplankton community composition and abundance depend intimately on mesoscale activity, we anticipate that ecosystem structure will be impacted by decreasing eddy kinetic energy in the subtropical gyres under climate warming (51). However, such a change will not be captured by Earth System Models that parameterize the mesoscale and phytoplankton community structure. These models used to derive climate projections do not explicitly resolve or differentiate laterally coherent and mixed waters, which we have shown are linked to distinct ecological outcomes. The lack of representation of diverse phytoplankton responses to specific types of mesoscale disturbances and dispersal could be a contributing factor as to why predicted declines in global primary production double when models are not eddy-resolving (52).

In summary, we found that eukaryotic phytoplankton are elevated in mesoscale eddies, along their edges, and in laterally mixed waters outside of eddies in the NPSG. By contrast, mesoscale outside-eddy water masses that experienced minimal exchange with surrounding waters in recent months were a sink for all major eukaryotic phytoplankton groups. We infer that these coherent waters isolate and transport a community sheltered within a progressively nutrient-depleted environment with reduced sources of replenishment. Outside of these circumstances, the bulk of the subtropical gyre is highly dispersive and eddying, sustaining small populations of eukaryotic phytoplankton in the face of competition with dominating cyanobacteria populations. These field observations support the theory that a combination of intermittent disturbance (53) and dispersal (20–23) fosters the coexistence of cyanobacteria and eukaryotic phytoplankton in the subtropical gyre.

### Data Archival.

The sequences from this project are available at NCBI Sequence Read Archive (BioProject ID: PRJNA1079727). Tables of volumetric gene abundance and taxonomic annotation are archived on Zenodo at https://doi.org/10.5281/zenodo.18867070 (54). The satellite velocity fields are distributed by CMEMS (29). The altimetric Mesoscale Eddy Trajectories Atlas (META3.2 DT) was produced by SSALTO/DUACS and distributed by AVISO+ https://aviso.altimetry.fr with support from CNES, in collaboration with IMEDEA https://doi.org/10.24400/527896/a01-2022.005 (3). This study utilizes the 10+ day lifespan eddy tracks from the allsat META3.2 DT product. Monthly average chlorophyll-*a* data for Fig. 1*A* was obtained from the Ocean Colour-CCI database (2).

## Materials and Methods

The scripts used for the data analysis and figure generation in this study are available at https://github.com/lexi-jones/G4_phyto_advection and archived on Zenodo at https://doi.org/10.5281/zenodo.20090970 (v1.0.0).

### Shipboard Sampling.

We filtered surface seawater samples in triplicate at 12 CCS sites, and 53 NPSG sites from the underway system (∼8 m depth) of the R/V Thompson during the November 2021 SCOPE Gradients 4 cruise (TN397). Samples were collected an average of 45.8 km apart (*SI Appendix*, Fig. S4*A*). At each site, one carboy was filled with ∼4 L of seawater, briefly mixed, and quickly separated into three 1 L bottles to serve as technical replicates. We filtered each 1 L replicate through 0.22μm Sterivex-GV filters (EMD Millipore; SVGVL10RC) with a peristaltic pump, without size-fractionating. The filters were sealed with Luer lock plug caps (MRO Supplies; 51525K333, 51525K334) and frozen at −80 ^°^C until DNA extraction.

### DNA Extraction, PCR, and Sequencing.

A detailed description of the protocol followed for DNA extraction and PCR can be found in ref. 26. In brief, 20μL of three genomic standards (*Blautia producta*, *Deinococcus radiodurans*, *Thermus thermophilus*) were spiked into 1.5 mL lysis buffer before DNA extraction. Cell lysis and DNA recovery protocols were adapted from refs. 55, 56, 57, including an additional bead-beating step. The DNA was amplified with the 515Y (59-GTGYCAGCMGCCGCGGTAA) and 926R (59-CCGYCAATTYMTTTRAGTTT) 3-domain universal primers (24, 25). We followed the wet lab procedures documented at doi.org/10.17504/protocols.io.vb7e2rn, except we used the GoTaq master mix (Promega, M5132/5133; Lot #0000520348) for amplification. Samples were sequenced at Tufts University Medical School with HiSeq RapidRun technology (2 × 250 bp).

### Genomic Annotation.

We demultiplexed the samples following github.com/jcmcnch/demux-notes and used a software pipeline developed for the primer set 515Y/926R for the ASV identification and taxonomic classification with SILVA 138.1 and PR2 4.14.0 (github.com/jcmcnch/eASV-pipeline-for-515Y-926R) (58–61). To correct for the known PCR bias against 18S sequences (59), we used an Agilent 2100 Bioanalyzer to quantify concentrations of amplicons in the sequencing pool, and the correction factor (=2.09) was derived by comparing the 16S:18S ratio of molarity. This correction factor was applied along with sample-specific adjustments to account for sequence loss during the quality control steps to merge the 16S and 18S reads into a single ASV table (github.com/fletchec99/normalizing_16S_18S_tags). The 16S rRNA reads were used to identify cyanobacteria and the eukaryotic phytoplankton chloroplasts, excluding Dinoflagellata because they have a unique organization of the chloroplast genome and may not have an amplifiable 16S rRNA gene (62). The dinoflagellate ASV abundances were instead estimated from the 18S rRNA reads. This study only included dinoflagellate ASVs with a known capacity for photoautotrophy (63, 64). In total, 3411 phytoplankton ASVs had nonzero abundance in at least one NPSG sample.

### Gene Abundances and Anomalies.

The 16S and 18S rRNA gene abundances were quantified as described in ref. 26, using the software from https://github.com/lexi-jones/internal_std_correction. In short, the absolute volumetric gene copy abundances for ASV i in sample j estimated from the genomic standard s is

[2] $$\begin{array}{c} A_{\mathrm{ijs}}=\frac{\left(R_{\mathrm{ij}}\;\cdot \;C_{s}\right)}{\left(R_{\mathrm{sj}}\;\cdot \;V_{j}\right)}, \end{array}$$

where $R_{\mathrm{ij}}$ is the number of reads, $C_{s}$ is the number of 16S rRNA gene copies of s (known a priori), $R_{\mathrm{sj}}$ is the number of reads of s, and $V_{j}$ is the volume of filtered seawater (65). Each of the three internal standards was used to obtain a unique estimate of the gene abundances of each ASV. We define the abundance of each ASV as the average of the replicates from triplicate sampling and three internal standards because the mean had the best fit to cyanobacteria cell abundances derived from flow cytometry (26).

The CCS samples were only used for contextual purposes in Fig. 1, with the remaining analysis conducted on the NPSG samples. To have sufficient information about the latitudinal and diel signals in abundance for the ASVs, we required that each contributed ≥0.01% total gene copy counts in at least one NPSG sample and nonzero at ≥10 to be included in this study. This left 406/3411 phytoplankton ASVs that contributed between 98.3 to 99.6% of the total phytoplankton gene copies from each site. The STA was computed for each of the 406 ASVs, following the procedure detailed in *SI Appendix*, *Spatiotemporal Anomaly*. 332/406 ASVs are eukaryotic phytoplankton, and the remaining cyanobacteria.

We performed a nonparametric bootstrap analysis to obtain 95% CIs for the STAs and the upstream results. For 10,000 bootstrap iterations, one replicate abundance measurement was randomly selected with replacement for each ASV and sample. The STAs, linear regressions, and exponential model fits were recomputed each bootstrap iteration to calculate 2.5 to 97.5th percentiles.

### Water Mass Coherence.

We initialized a Gaussian distributed cloud of 1000 Lagrangian particles around each sample site with a SD of $0.025^{{}^{\circ}}$ from the central location. We performed this analysis in triplicate, and the three initializations were consistently used at each site. The maximum particle distances from the actual sample location for each run were 9.4, 10.0, and 10.4 km. The Lagrangian trajectories were simulated in daily, $1/4^{{}^{\circ}}$ satellite geostrophic velocity fields (29) with the Python package OceanParcels (v2.2.2) (66). We used a 4th-order Runge–Kutta advection scheme to compute particle locations every 20 min and saved them every 6 h. The particle cloud was considered coherent until the mean displacement was twice the initialization distance from the center of mass. Sensitivity tests were performed to determine this threshold, which was selected based on the displacement after which the particles tended to increasingly diverge in their advective pathways. 95% of runs in the NPSG were within 3.99% of the mean coherence time measured at the respective sites, so the coherency metric varied minimally between the triplicate runs (Fig. 3). The coherence times presented in this study are the means of the triplicates.

To estimate the fraction of water masses across the NPSG that were contained in an outside-eddy coherent water mass during the sampling campaign, we mimicked the Gaussian cloud calculation in a less computationally expensive manner. We conducted a simulation seeding particles at $1/50^{{}^{\circ}}$ gridded resolution on November 23, 2021, outside of eddies and eddy edges, but within the dashed polygon region of Fig. 1*A*. A coherence time was calculated for 7 × 7 clusters of gridded particles (removing the corner particles, see *SI Appendix*, Fig. S11*A*). The coherence time was computed as a weighted mean of the particle spread from the center, again to mimic the particle distribution of the Gaussian clouds. We conducted sensitivity tests to choose the $1/50^{{}^{\circ}}$ resolution, finding this was the coarsest initialization that reasonably correlated with the coherence times measured from the high-density Gaussian cloud simulations from the sample sites (*SI Appendix*, Fig. S11*B*). The gridded estimates are biased toward shorter coherence times compared to the clouds, and thus, the gyre-scale estimates (*SI Appendix*, Fig. S11*D*) are conservative.

## Supplementary Material

Appendix 01 (PDF)

Dataset S01 (CSV)

Movie S1. 150-day backward Lagrangian trajectories of the Gaussian clouds initialized at each sample site. Outside-eddy samples have a gray color scheme, cyclones blue, and anticyclones red. Sample cites are labeled with a circle if outside an eddy, a square for inside, and a triangle on eddy edges (< 15km from the boundary). The background is the contours of the mean absolute dynamic topography.

## Data Availability

Derived data (54) and scripts (67) generated for this analysis are archived on Zenodo. The sequence data is available on NCBI BioProject (68). The satellite velocity fields are distributed by CMEMS (29). The altimetric Mesoscale Eddy Trajectories Atlas (META3.2 DT, allsat) was produced by SSALTO/DUACS and distributed by AVISO+ with support from CNES, in collaboration with IMEDEA (3). This study utilizes the 10+ day lifespan eddy tracks. Monthly average chlorophyll-a data for Fig. 1*A* was obtained from the Ocean Colour-CCI database (2).
