# Supplementary material for: The dynamic mesoscale sink and source niches for eukaryotic phytoplankton in a subtropical gyre
Source: Proc Natl Acad Sci U S A. 2026 Jun 17;123(25):e2608700123. doi: 10.1073/pnas.2608700123 (PMC13291661; doi:10.1073/pnas.2608700123)
Supplement: Supplementary file 1 — Appendix 01 (PDF) [file pnas.2608700123.sapp.pdf]

Supporting Information for

The dynamic mesoscale sink and source niches for eukaryotic phytoplankton in a subtropical gyre

Alexandra E. Jones-Kellett, Jesse C. McNichol, Yubin Raut, Jed A. Fuhrman, and Michael J. Follows

Alexandra E. Jones-Kellett
E-mail: aejk@alum.mit.edu

This PDF file includes:

Supporting text
Figs. S1 to S12
Tables S1 to S4
Legend for Movie S1
SI References

Other supporting materials for this manuscript include the following:

Movie S1

Supporting Information Text

Spatiotemporal Anomaly

The spatiotemporal anomaly (STA) for each ASV with volumetric 16S or 18S gene abundance A (copies/mL) was computed as follows:

1. **Latitudinal Rolling Mean:** We first applied a correction by universally adding 1 gene copy/mL to all ASV abundances to avoid division by zero in Step 2. This is what we refer to as A in the proceeding pipeline. To account for the latitudinal trend in abundance, a centered rolling mean was calculated as

$$\mathrm{RM}(x_\ell) = \frac{1}{9}\sum_{i=-4}^{4} A(x_{\ell+i}) \qquad [1]$$

such that 9 is the window size centered on the latitudinal position of the sample x_ℓ. We use the subscript ℓ to refer to the latitude-based index, i.e., the sequential order of the original sample collection. A window size of 9 includes samples approximately 183.2 km on either side of the center sample, based on the mean spatial separation of 45.8 km between neighboring samples (Fig. S4a). This was implemented in Python with the `pandas` package (Version 1.3.3; https://doi.org/10.5281/zenodo.5501881):

```
# df is a pandas DataFrame with latitudinally sorted samples as rows & ASVs as columns
df.rolling(window=9,center=True,min_periods=4).mean()
```

The minimum periods parameter choice allows the rolling mean to be computed at the beginning and end of the transect, with the rolling mean window decreasing in size given the available data. For example, the rolling mean at the first index is computed based on the following 4 samples and the previous 4 samples for the last index.

2. **Normalized Latitudinal Anomaly:** We subtracted the latitudinal rolling mean from the original abundance and then normalized by the rolling mean,

$$\mathrm{nLA}(x_\ell) = \frac{A(x_\ell) - \mathrm{RM}(x_\ell)}{\mathrm{RM}(x_\ell)} \qquad [2]$$

3. **Temporal Sort:** The local time since sunrise was computed based on the sample latitude, longitude, and collection time with the `suntime` Python package (Version 1.3.2; https://github.com/SatAgro/suntime). $\mathrm{nLA}(x_t)$ was derived from $\mathrm{nLA}(x_\ell)$, letting x_t denote the time to the closest sunrise for the samples, with the t subscript referring to a re-indexing of the samples based on the time since sunrise.

4. **Temporal Rolling Mean:** Unlike the spatial trends, the temporal trends in ASV abundance are cyclic, or "looping", so we computed a looping rolling mean from the temporal sort. An additional consideration was that samples were collected at arbitrary times based on estimates of equidistant spatial separation. While the coefficient of variation (ratio of the standard deviation to the mean) is only 9.53% for the spatial separation between samples, it is 79.8% for the temporal separation (Fig. S4). To account for the temporal irregularity, we weighted the samples within the rolling mean window with an exponential decay function based on the temporal distance from the center sample.

The looping, weighted temporal rolling mean was computed with a window size of 7, centered on sample x_t. This includes samples collected within approximately 64.8 minutes of the center sample, based on the mean spatial separation of 21.6 minutes between neighboring samples (Fig. S4b). For each window, the unnormalized weights are

$$w_{ti} = \exp\left(-\frac{|x_{(t+i) \bmod T} - x_t|}{D_t}\right) \qquad [3]$$

where $D_t = \max(|x_{(t+i) \bmod T} - x_t|)$ for $i \in \{-3,-2,-1,0,1,2,3\}$, or the maximum temporal distance to x_t within the window. $T = 53$ is the total number of data points, and the modulo operation enables looping indexing, wrapping the windows around the endpoints. We then normalized the weights by their sum,

$$\tilde{w}_{ti} = \frac{w_{ti}}{\sum_{j=-3}^{3} w_{tj}} \qquad [4]$$

and computed the weighted looping rolling mean,

$$\mathrm{RM}(x_t) = \sum_{i=-3}^{3} \tilde{w}_{ti} \cdot \mathrm{nLA}(x_{(t+i) \bmod T}) \qquad [5]$$

This was executed with the following Python function using the `pandas` (pd; Version 1.3.3) and `numpy` (np; Version 1.21.2) packages:

```python
def rolling_mean_looping_weighted(df, x_values, window):
    """
    Compute a weighted rolling mean with (looping) periodic boundary conditions,
    weighted by the distance between x-values. The window is centered on the center value.

    Params
    - df: pandas DataFrame to apply the rolling mean
    - x_values: x-values associated with the rows of df (e.g., time since sunrise)
    - window: window size (e.g. window=7 will be 3 on either side of center point)

    Returns
    - A df with the weighted rolling mean applied
    """
    # initialize a dataframe
    result = pd.DataFrame(index=df.index, columns=df.columns, dtype=float)

    # Iterate through each ASV
    for col in df.columns:
        rolling_mean_column = []

        # Iterate through the samples to compute looping rolling mean
        for i in range(len(df)):

            # Define current the window
            inds = [(i + j - window // 2) % len(df) for j in range(window)] # window indeces with looping
            window_vals = df.iloc[inds, df.columns.get_loc(col)].values
            x_window = x_values.iloc[inds].values # window x's
            x_current = x_values.iloc[i] # center x
            distances = np.abs(x_window - x_current) # distance between the current point and other vals in the window
            normalized_distances = distances/np.max(distances)

            # Exponential decay for weights based on distance
            weights = np.exp(-normalized_distances)
            normalized_weights = weights/weights.sum()

            # Compute the weighted mean
            rolling_mean_column.append(np.sum(window_vals*normalized_weights))

        result[col] = rolling_mean_column

    return result
```

5. **Spatiotemporal Anomaly:** We then subtracted the temporal rolling mean from the normalized latitudinal anomaly

$$\text{nSTA}(x_t) = \text{nLA}(x_t) - \text{RM}(x_t) \quad [6]$$

yielding a spatiotemporal anomaly that is still normalized by the original ASV abundance. Finally, we re-indexed the data by the original latitudinal sort and unnormalized by multiplying the latitudinal rolling mean to obtain the final STA used in the study,

$$\text{STA}(x_\ell) = \text{nSTA}(x_\ell) \cdot \text{RM}(x_\ell) \quad [7]$$

Vizualizations of the STA calculation for various phytoplankton ASVs are provided in Fig. S2 and in the Jupyter notebook at https://github.com/lexi-jones/G4_phyto_advection/blob/master/lat_diel_running_means_v3.ipynb.

Justification of Geostrophic Approximation

Our results interpret the biological response to the flow assuming the geostrophic approximation, i.e., a balance between the Coriolis force and the pressure gradient force. Importantly, the geostrophic approximation does not incorporate wind-driven components of the surface flow. The frictional boundary layer at the ocean-atmosphere interface includes an Ekman spiral that decays with depth (1) and an average Ekman transport. Ekman transport in the open ocean is a short-term forcing due to the transient nature of wind events, whereas geostrophic flow structures persist over long time scales. Nevertheless, the seawater samples used in the study were collected in the surface ocean (∼8m depth) where Ekman flows have the strongest velocities and may be important.

Figure S7 provides a visual comparison of equivalent Lagrangian particle trajectories initialized as Gaussian clouds from the study sample sites. The particles were advected in daily, 1/4° CMEMS geostrophy (2) and 1/4° OSCAR geostrophy+Ekman (3) satellite velocity fields using the same parameter choices. The OSCAR surface currents represent the total flow averaged over the upper 30m of the water column. We found that the backward in time trajectories in the geostrophic flow originate from a variety of directions, whereas the geostrophy+Ekman velocities suggest that with sufficient time waters originated from the southeast for nearly every sample site. The Ekman-included trajectories also exhibited higher transport distances from the southern samples compared to the northern. Physical tracers such as temperature, which is more conservative than biological tracers, could indicate which are the relevant transport pathways for the spatial and temporal domain of interest.

We used underway measurements of sea surface temperature (SST) to test the relevance of the derived Ekman flow on tracer transport for months. First, we computed the STA of SST in the same manner as the ASV abundances, except the latitudinal anomaly was not normalized (Fig. S6). The SST STA is the residual value subtracting the latitudinal increase in temperature as the ship transited southward, and the diel cycle of heating and cooling. In the portion of the transect exhibiting a linear relationship between temperature and latitude, one would expect SST anomalies to be dependent on the meridional (i.e., north/south) transport. To test if this was the case, we identified the largest segment of the SST latitudinal rolling mean with a linear fit that exhibits an $R^2 >= 0.95$. This segment includes most samples, excluding only a few at the beginning and end (Fig. S5(a)). We then compared the SST STA of the linear segment to the distance north that the water masses originated, computed from each of the two velocity products. Assuming temperature is mostly a conservative tracer, then the SST STA is expected to decrease as a function of the distance north, where it is colder. We found that within 60 days, the SST STA has a significant linear relationship with the water mass's origin distance north for both geostrophy and geostrophy+Ekman flow fields (Fig. S5b,c). The Ekman-included flow has a slightly more significant relationship with temperature anomalies for this shorter timescale. For timescales longer than 60 days, the trajectories from the geostrophy-only flow fields have a more significant relationship with SST. After 90 days, the geostrophy+Ekman flow does not have a significant linear relationship with the distance north.

Our findings that temperature transport by Lagrangian trajectories simulated in Ekman-included flow fields is significant for short time scales aligns with the transient nature of wind-driven events. But, for the timescales of interest in the main study (several months), we conclude that geostrophy is the dominant component of the flow relevant for studying the transport of biological communities in this domain. Furthermore, we did not find any relationships between the phytoplankton communities and Lagrangian trajectories computed from the geostrophy+Ekman product. This suggests that the geostrophic component of the flow is the leading-order contribution to the mesoscale advection of populations in the NPSG, especially for timescales ≥60 days. We note, however, that the accuracy of the Lagrangian trajectories depend on the accuracy of the input velocity fields. The geostrophic product is subject to less processing and model bias than the Ekman-included flow, so the results could also be indicative of error propagation in the geostrophy+Ekman velocity fields.

Tracer Equation Scaling

We interpret the change in eukaryotic phytoplankton populations in coherent water masses with an advection-diffusion equation in the Lagrangian reference frame. We start with the Eulerian, temporal rate of change in concentration of a tracer C (e.g., the volumetric gene copies of eukaryotic phytoplankton) in a fluid

$$\frac{\partial C}{\partial t} = \underbrace{-\nabla \cdot (UC)}_{\text{advection}} + \underbrace{\nabla \cdot (\kappa \nabla C)}_{\text{eddy diffusion}} + \underbrace{\mu C}_{\text{net growth}} \quad [8]$$

where U is the three-dimensional velocity vector, κ is the tensor of eddy-diffusion coefficients representing the effects of unresolved flows, and μ is the net growth rate. We transform the equation into the Lagrangian frame following the horizontal flow (subscript H), $\frac{D_H C}{Dt} = \frac{\partial C}{\partial t} + u\frac{\partial C}{\partial x} + v\frac{\partial C}{\partial y}$, and integrate over the depth of the mixed-layer, Z_{ML}, within which properties are assumed to be vertically homogeneous. This assumes the absence of strong external drivers of vertical mixing, supported by the minimal atmospheric input of turbulent kinetic energy and shallow, uniform mixed-layer depths in the region over the summer months preceding sampling (see next section "Turbulent Kinetic Energy and Mixed Layer Depth" and Fig. S12). We consider the idealized situation where Z_{ML} is unchanging in space and time, and there is no horizontal shear within the mixed-layer, leading to an equation for the rate of change of properties in the mixed-layer column following the horizontal flow:

$$\frac{D_H\ C}{Dt} = \kappa_H \left(\frac{\partial^2 C}{\partial x^2} + \frac{\partial^2 C}{\partial y^2} \right) - \frac{1}{Z_{ML}} \left([wC]_{Z_{ML}} - \kappa_Z \left[\frac{\partial C}{\partial z} \right]_{Z_{ML}} \right) + \mu C \quad [9]$$

We make the following scaling argument of Eq. 9:

$$\frac{\Delta C}{\Delta t} \sim \kappa_H \frac{\Delta C}{\Delta x^2} + \kappa_H \frac{\Delta C}{\Delta y^2} - \frac{w \Delta C}{Z_{ML}} - \frac{\kappa_Z \Delta C}{Z_{ML}^2} + \mu \Delta C \Rightarrow \frac{1}{\Delta t} \sim \frac{\kappa_H}{\Delta x^2} + \frac{\kappa_H}{\Delta y^2} - \frac{w}{Z_{ML}} - \frac{\kappa_Z}{Z_{ML}^2} + \mu$$

where Δy is the horizontal mixing length and Δt is the timescale of interest. We let $\Delta t \sim 1.3 \times 10^{-7}\ \mathrm{s}^{-1}$ (= 90 days). The third term on the right is an order of magnitude smaller, assuming $w = 10^{-6}$ m/s (4) and $Z_{ML} = 50$m (Fig. S12). The fourth term on the right is two orders of magnitude smaller, assuming $\kappa_Z \sim 10^{-5}\mathrm{m/s}^2$ (5). The scaling argument simplifies Eq. 9 to

$$\frac{1}{C}\frac{D_H C}{Dt} \sim \frac{\kappa_H}{C} \left(\frac{\partial^2 C}{\partial x^2} + \frac{\partial^2 C}{\partial y^2} \right) + \mu \quad [10]$$

Empirically constrained power-law relationships of lateral eddy-diffusion at the appropriate scale suggest that the first term on the right in Eq. 10 can be significant (6), although its sign will depend upon the second derivative of the population density in space, i.e., lateral diffusion may drive population gain or loss. Here we explore the limit where lateral diffusion may be neglected:

$$\frac{1}{C}\frac{DC}{Dt} \sim \mu \quad [11]$$

The solution to Eq. 11 is an exponential decay function,

$$C(t) = C_0 e^{\mu t} \quad [12]$$

where C_0 is the initial population size.

Turbulent Kinetic Energy and Mixed Layer Depth

To indicate the potential influence of meteorologically forced vertical mixing during the months up to and including the sampling dates, we evaluated the Turbulent Kinetic Energy (TKE, $m^3 s^{-3}$) input to the surface mixed layer. Wind stress on the ocean surface and heat (buoyancy) loss both introduce energy that drives and is dissipated by small-scale turbulence with impacts including the thickening of the mixed layer (7, 8). In turn, this can lead to entrainment of thermocline waters that are rich in nutrients and/or dilution of surface plankton populations. Therefore, quantifying these sources of TKE provides a diagnostic for potential biogeochemical effects of atmospheric forcing. We evaluate TKE input following (9):

$$\int_{-h}^{0} \frac{d(TKE)}{dt} dz = m_1 u_*^3 - m_2 \frac{\alpha g}{\rho^2 C_p} \frac{h}{2} H_o \quad [13]$$

where h is the mixed layer thickness. The first term on the right represents an input of TKE from wind stress on the ocean surface where the friction velocity $u_* = (|\tau|/\rho)^{0.5}$ and $|\tau|$ (N m^{-2}) is the magnitude of the total surface wind stress. $m_1 = 1.25$ is a dimensionless coefficient. The second term on the right represents the source of turbulent mixing energy when the ocean is cooling, driving convective mixing, or acting as a sink of TKE when ocean heat gain is driving restratification. Here ρ is the density of seawater (kg m^{-3}), C_p (J kg^{-1} K^{-1}) is the heat capacity of seawater, α (kg m^{-3} K^{-1}) is the thermal expansion coefficient of seawater, g (m s^{-2}) is the constant of gravitational acceleration, and H_o (W m^{-2}) is the net heat flux across the sea surface. When the ocean is losing heat ($H_o < 0$), $m_2 = 1.0$ and there is a buoyancy-driven source of TKE; when the ocean is gaining heat ($H_o > 0$), $m_2 = 0.2$ and there is a buoyancy-driven sink of TKE.

We evaluated the TKE input to the ocean, as formulated above, over the North Pacific sample domain for the calendar years 2020 and 2021. We used the midnight UTC, hourly-averaged data from the ECMWF ERA5 reanalysis at https://doi.org/10.24381/cds.adbb2d47 (10) for wind-stress and heat flux data on a 0.25° lat-long grid. The net heat flux, H_o = latent heat + sensible heat + long-wave radiation + short-wave radiation. Monthly 0.25° mixed layer depth (h) was taken from the ECMWF ORAS5 reanalysis at https://doi.org/10.24381/cds.67e8eeb7 (11). The data was re-gridded to align with the ERA5 products, and we used the contemporary monthly-averaged values of h for each daily evaluation of TKE input. These data products are used in Fig. S12.

Lagrangian Eddy Trapping

We initialized Lagrangian particles in uniform, 1/32° grids for each day of the cruise, and calculated their trajectories for 28-days backward-in-time in daily, 1/4° satellite geostrophic velocity fields. These simulations were used to calculate daily Lagrangian Averaged Vorticity Deviations (LAVD) (12), which is used as a measure of eddy trapping. For each Lagrangian particle X,

$$LAVD_{t_0}^{t_1}(X) = \frac{1}{|t_1 - t_0|} \int_{t_0}^{t_1} |\zeta(X(t)) - \overline{\zeta(t)}| dt \quad [14]$$

where $\zeta(X(t))$ is the relative vorticity of the particle at time t, $\overline{\zeta(t)}$ is the average relative vorticity across the spatial domain at time t, and $[t_0, t_1]$ is the temporal domain of the Lagrangian simulation. The LAVD field for each sampled eddy is shown in Fig. S10.

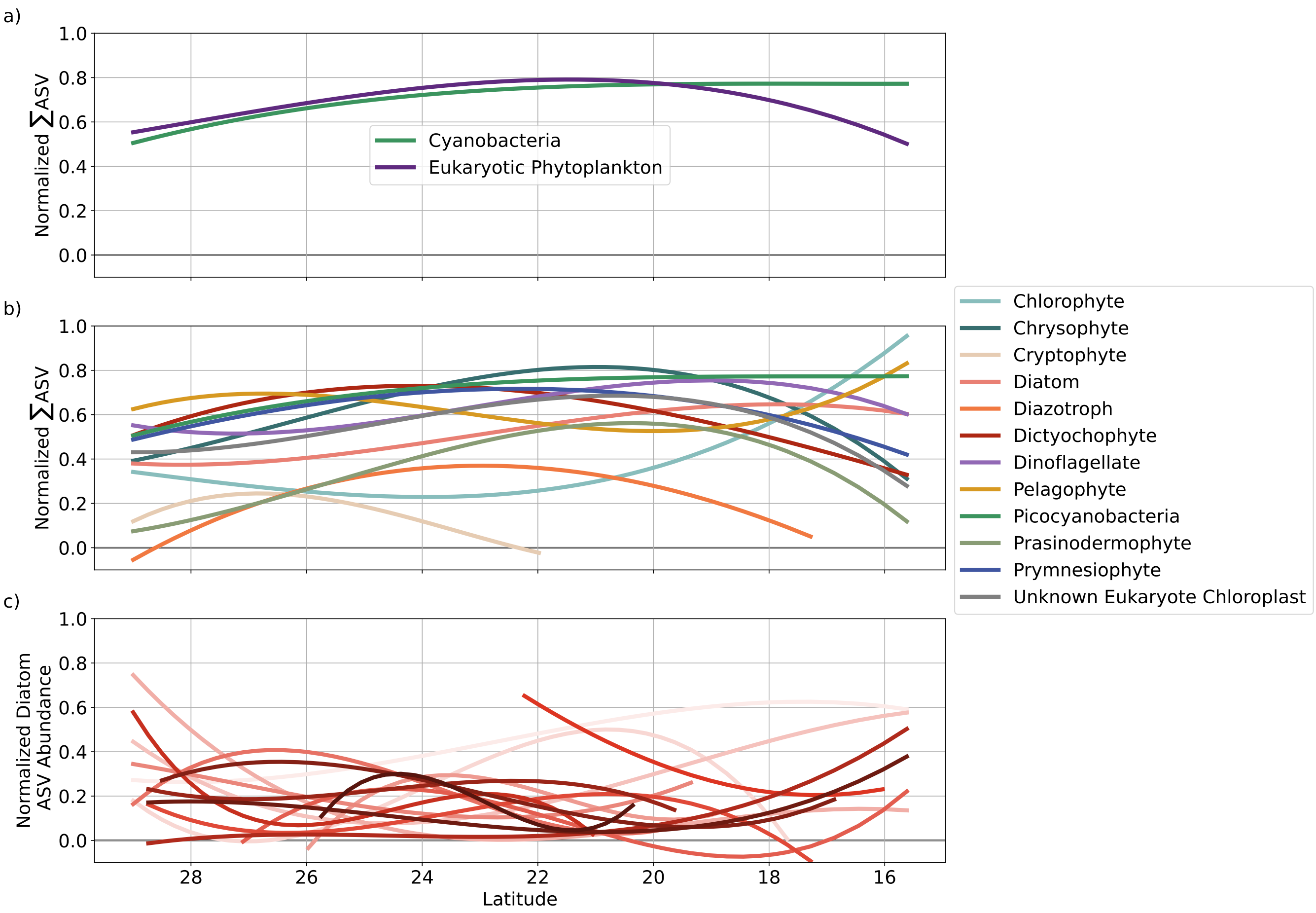

Fig. S1. Latitudinal trends in gene abundance at the **a)** domain, **b)** eukaryote taxonomic group (class or phylum), and **c)** ASV levels (showing 16 diatom ASVs). The abundances were normalized and fit with a 3rd-degree polynomial, ignoring trailing 0 abundances. This data is organized latitudinally from north to south, in congruence with the data collection.

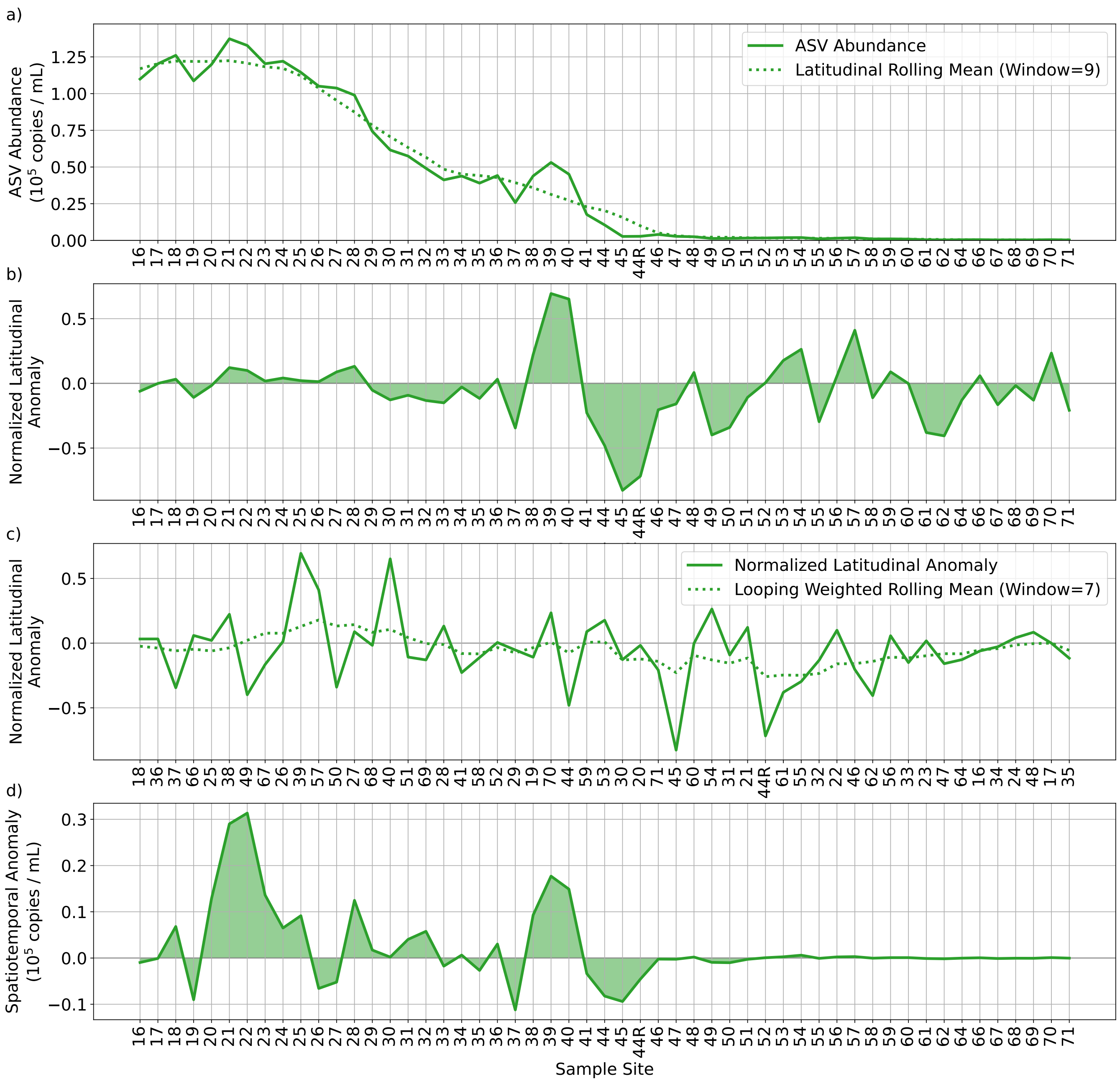

Fig. S2. An example of the steps to derive the spatiotemporal anomaly of gene abundance for a *Prochlorococcus* ASV (86cd70d2450e3f44f4a7c543f53008ee). **a)** The solid line represents volumetric gene abundance in the sample sites organized latitudinally, from north to south. The dotted line shows the latitudinal rolling mean computed with a window size of 9. **b)** The normalized latitudinal anomaly organized by latitude. **c)** The normalized latitudinal anomaly sorted by the local time since sunrise for each sample. The dotted line shows the looping weighted rolling mean computed with a window size of 7. This ASV has a daily cycle in abundance, peaking in the morning and minimizing in the evening. **d)** The spatiotemporal anomaly (STA), sorted latitudinally.

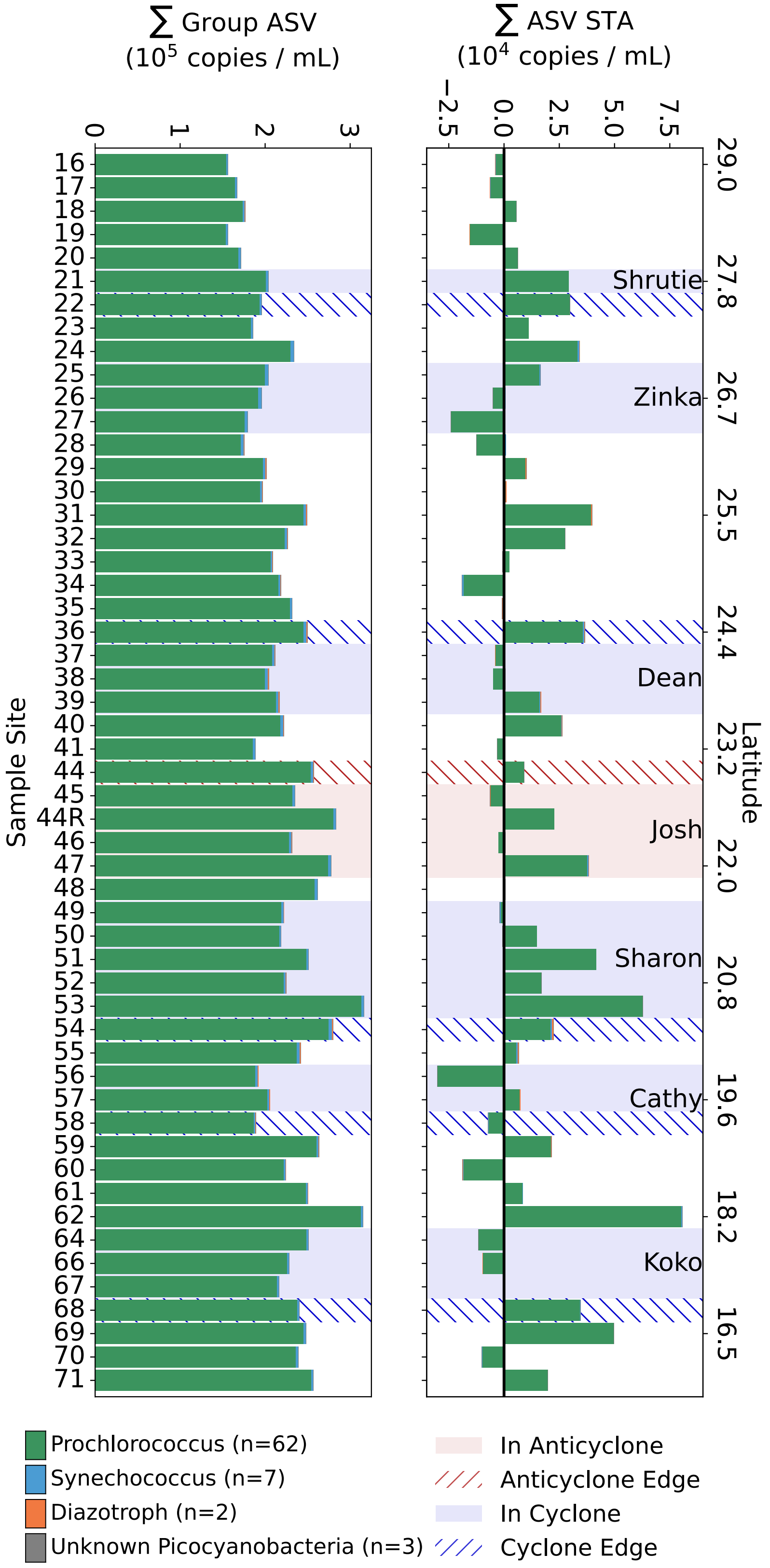

Fig. S3. Cyanobacteria stacked ASV abundances (left) and STAs (right). Note that the abundances on the left are an order of magnitude larger than the STAs. The blue and red shading delineates cyclonic and anticyclonic eddy regions, respectively. The hashed samples are eddy edge. The legends include information about the number of ASVs in each taxonomic group (n=x).

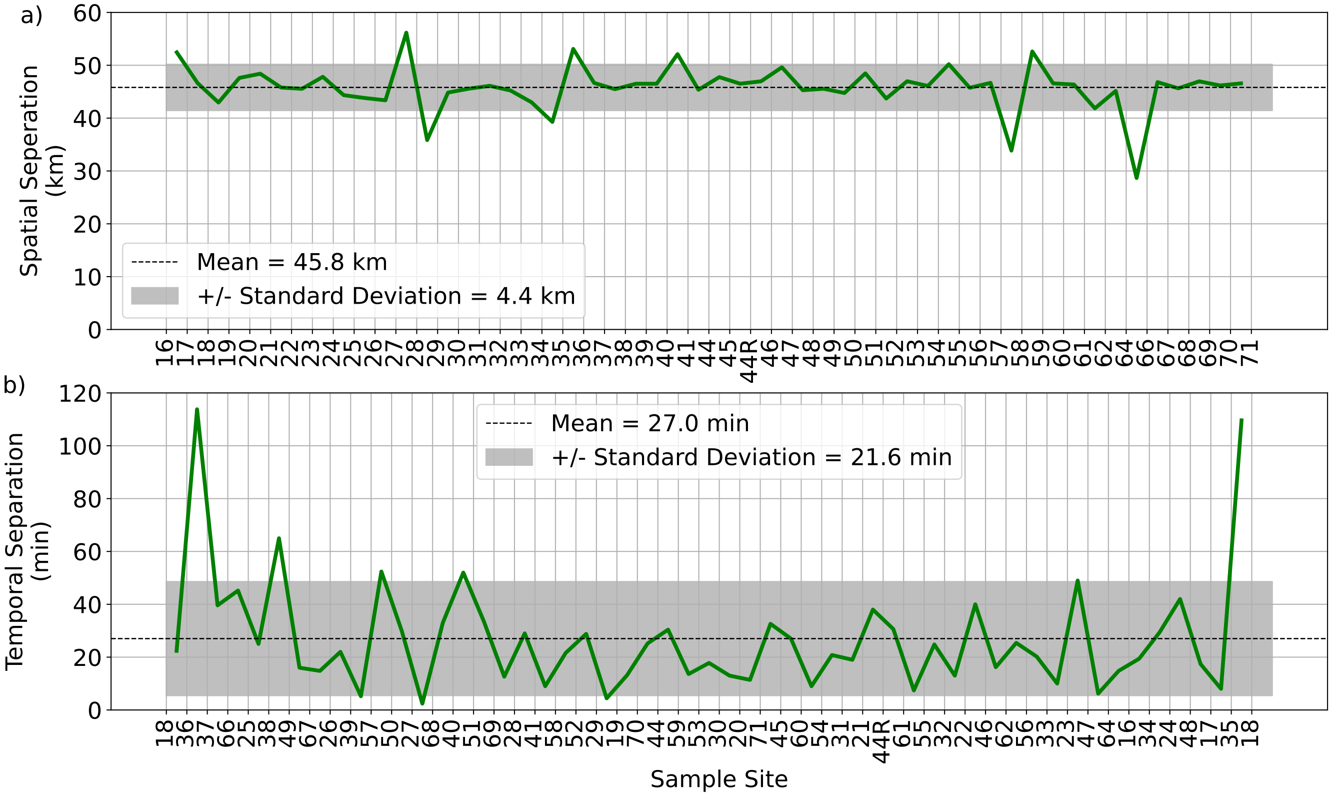

Fig. S4. Spatiotemporal distances between the sample sites. **a)** The spatial separation between neighboring sample sites. The coefficient of variation (ratio of the standard deviation to the mean) is 9.53%. **b)** The temporal separation between samples sorted by time to sunrise. This includes a looping between the first and last samples (i.e., sample 18 is at the beginning and end). The coefficient of variation is 79.8%.

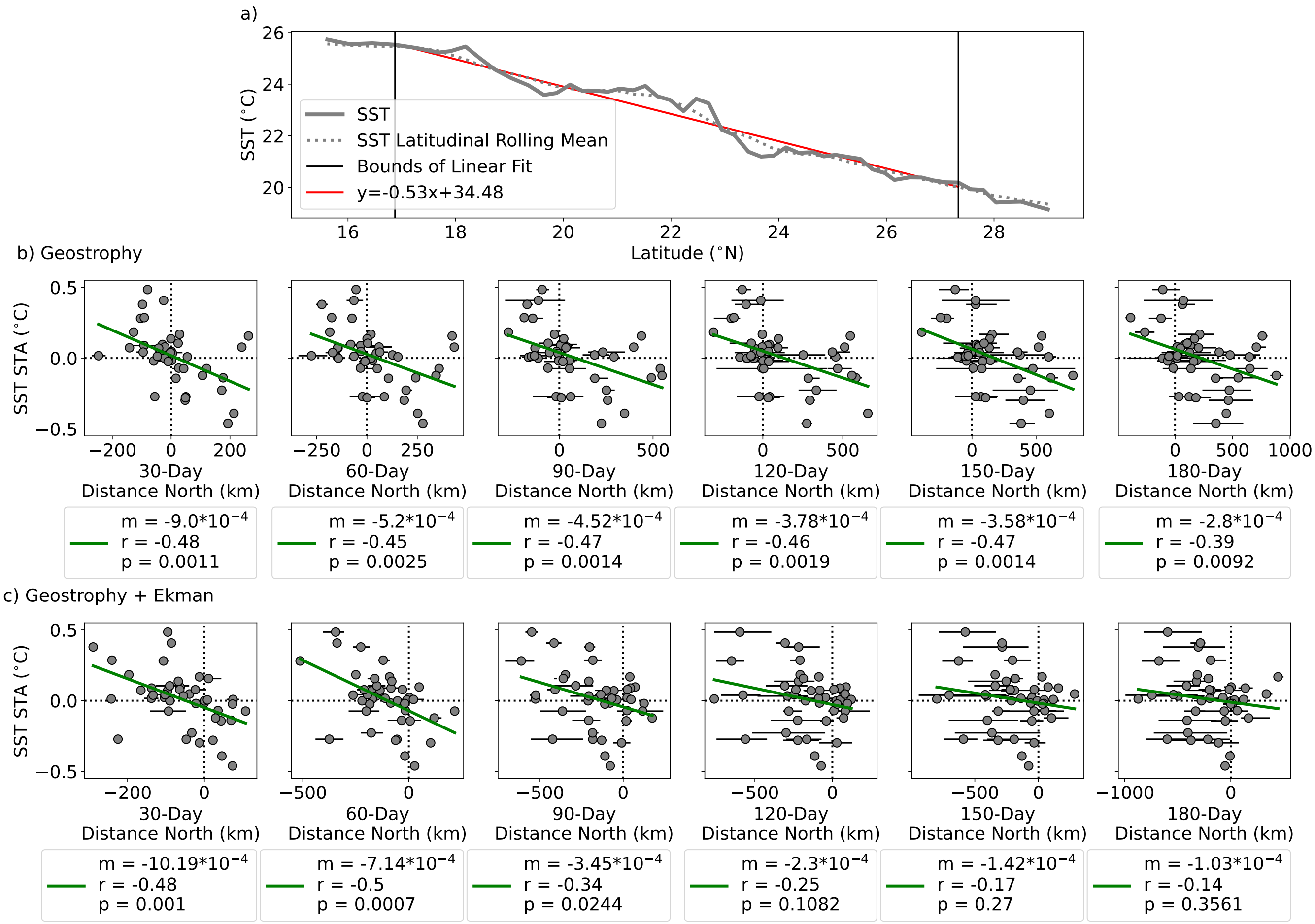

Fig. S5. Underway sea surface temperature (SST) and its relation to the water mass advection histories. **a)** SST along the sampling transect and the latitudinal rolling mean. The linear segment was chosen as the maximum window for a linear fit (red line) to the latitudinal rolling mean with an $R^2 \geq 0.95$. **b)** SST STA as a function of the Gaussian particle cloud origin distances north monthly for up to 180 days backward in time. Fig. S6 shows the transformation of the data from SST to an STA. Negative distances north suggest that the clouds originated from latitudes south of the sample site. The scatter plots include data within the linear segment of panel (a), marked with the vertical black bounds. **c)** Same as (b) but with the OSCAR geostrophy+Ekman satellite velocity product (3). A visualization of the difference in particle trajectories for the geostrophy and geostrophy+Ekman products is provided in Fig. S7.

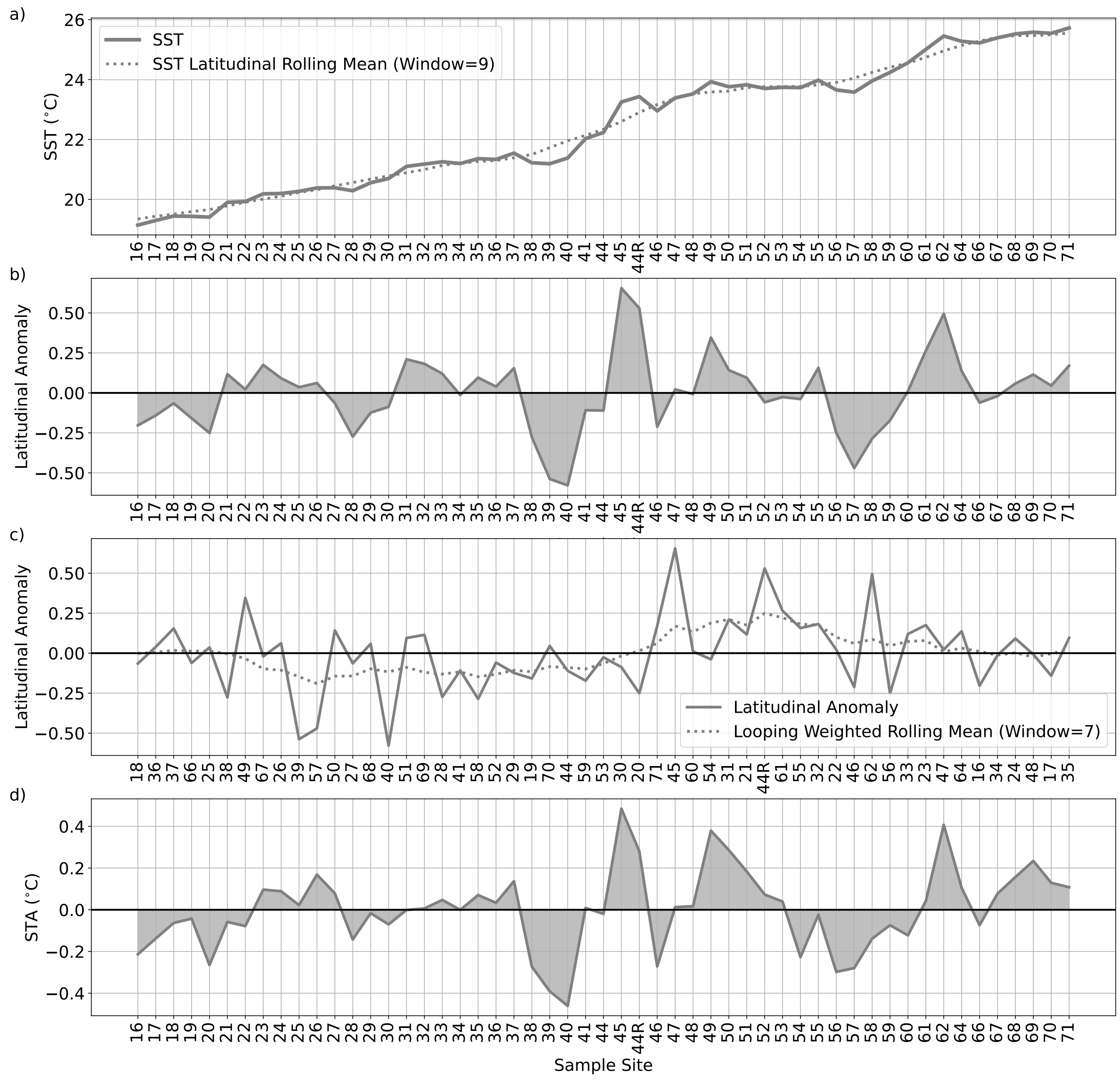

Fig. S6. The steps to derive the spatiotemporal anomaly of SST. This computation parallels the one performed to compute the biological STAs, except the latitudinal anomaly is not normalized. **a)** The solid line represents SST in the sample sites organized latitudinally, from north to south. The dotted line shows the latitudinal rolling mean computed with a window size of 9. **b)** The latitudinal anomaly, organized by latitude. **c)** The latitudinal anomaly, sorted by the local time since sunrise for each sample. The dotted line shows the looping weighted rolling mean computed with a window size of 7. This shows the daily cycle of heating and cooling, with temperature at a minimum in the morning and a maximum in the evening. **d)** The spatiotemporal anomaly (STA), re-sorted latitudinally.

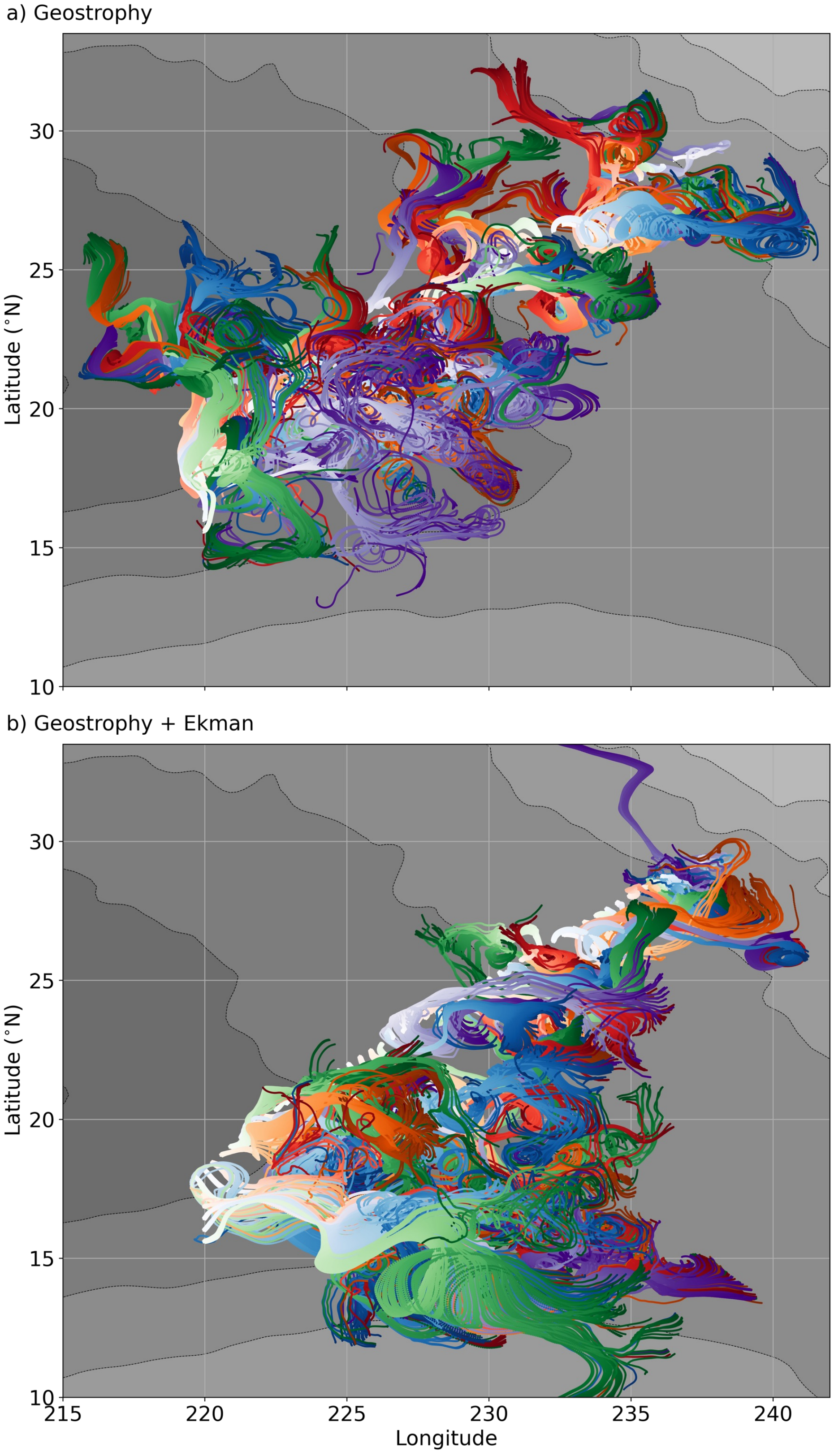

Fig. S7. 150-day backward-in-time Lagrangian trajectories of particles initialized as Gaussian clouds at the sample sites. **a)** Trajectories simulated in daily, $1/4^{\circ}$ geostrophic flow fields (2). The color palettes were cycled through to distinguish the trajectories from differing sample sites. The background is the contours of the mean absolute dynamic topography. **b)** Trajectories simulated in daily, $1/4^{\circ}$ geostrophic+Ekman flow fields, modeled by the OSCAR project (3). The majority of the particles originated from the southeast direction over the 5-month time frame.

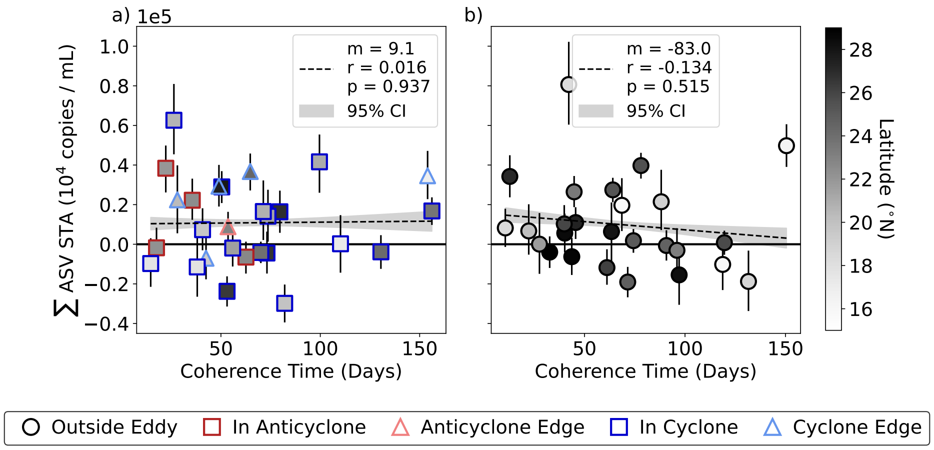

Fig. S8. Cyanobacteria STAs as a function of coherence time. **a)** Eddy-associated samples, with blue outlines corresponding to cyclones, and red outlines to the anticyclone. The triangle shape indicates an eddy edge sample, while the squares indicate in-eddy. The interiors of the scatter points are colored by latitude. m is the slope of the linear fit, r is the Pearson correlation coefficient, and p is the p-value. The gray shaded region shows the 95% confidence intervals (CIs) for the regression. The vertical black lines on each scatter point show the 95% CIs for the STA values. **b)** Same as (a) but for outside-eddy samples.

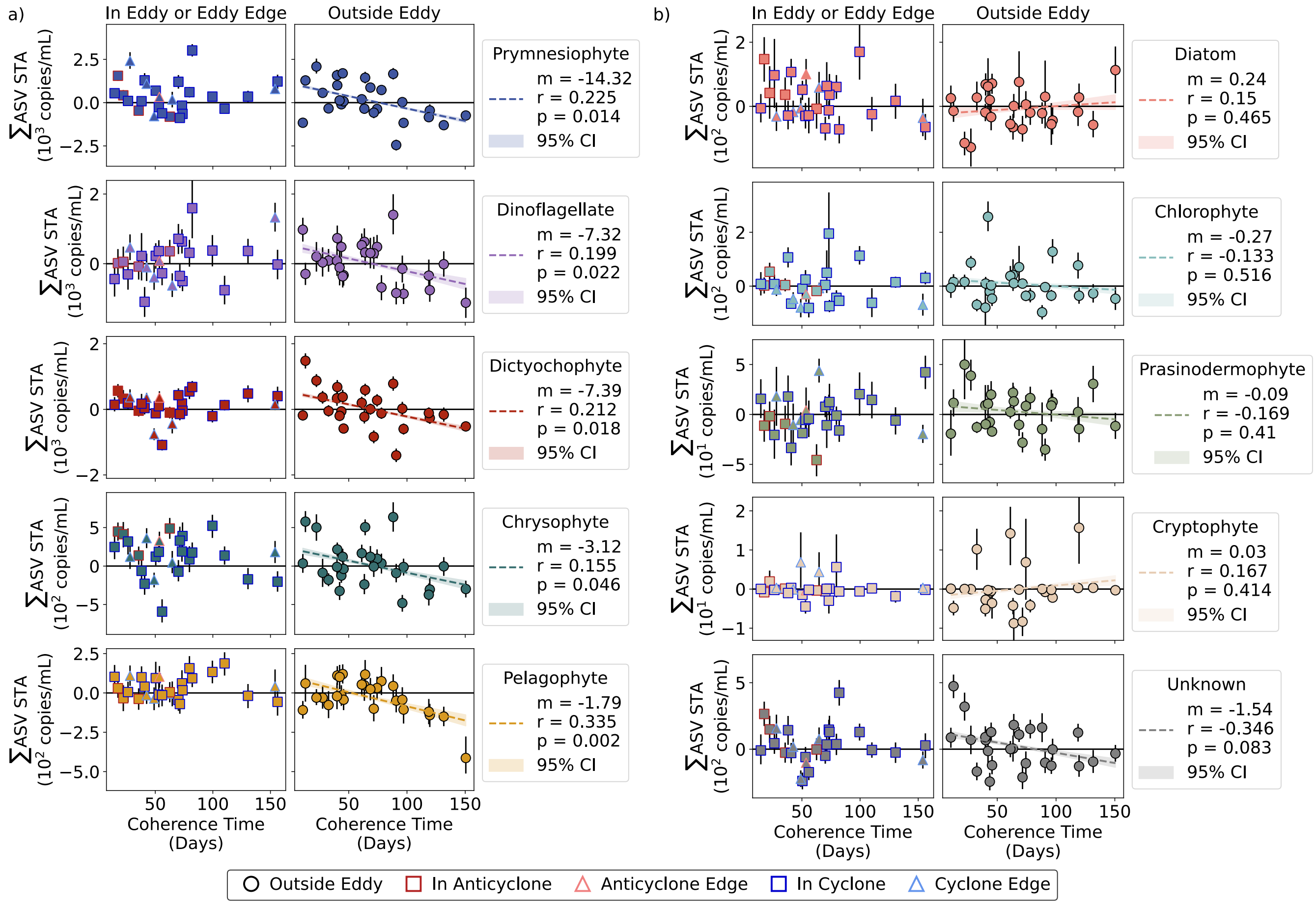

Fig. S9. The sum of eukaryotic phytoplankton ASV STAs at the group level compared to the water mass coherence time. **a)** The most abundant eukaryote groups. Eddy-associated samples are on the left, and outside-eddy samples are on the right. All of these abundant groups have a statistically significant linear relationship ($p < 0.05$) between the STA and coherence time outside of eddies, but not inside. The shaded region shows the 95% CIs for the regression. The vertical black lines on each scatter point show the 95% CIs for the STA values. **b)** Same as (a), but the rare groups and unknown eukaryotes. None exhibits a statistically significant relationship between the STA and coherence time, in or out of eddies.

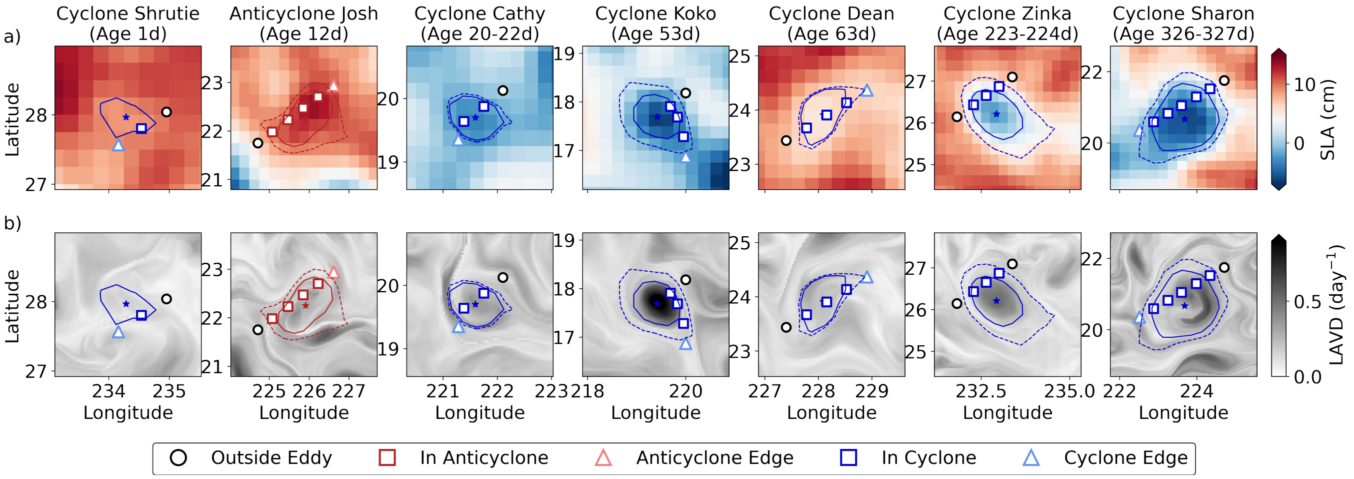

Fig. S10. A zoom-in on the internal structures of each mesoscale eddy, sorted by their age upon crossing (Table S2). **a)** The satellite Sea Level Anomaly (SLA) is plotted in the background. The solid line depicts the eddy speed contour, or the eddy's maximum circum-average geostrophic speed. The dotted line is the effective contour, or the largest closed contour surrounding the local maxima/minima in sea surface height. We define the eddy boundary as the effective contour in this study. The stars indicate the eddy centers, i.e., the local SLA extrema. The samples collected in an eddy are labeled with squares. One sample outside the eddy on either side of the boundary is shown here, with the triangles indicating eddy edge ($<$15 km from the effective contour), and the circle samples were collected ≥ 15 km from the boundary. **b)** The 28-day backward in time Lagrangian Averaged Vorticity Deviation (LAVD; Eq. 14), a measure of eddy trapping. Only Cyclone Koko contained a persistent Lagrangian coherent vortex, but samples were collected on the edge of the coherent structure.

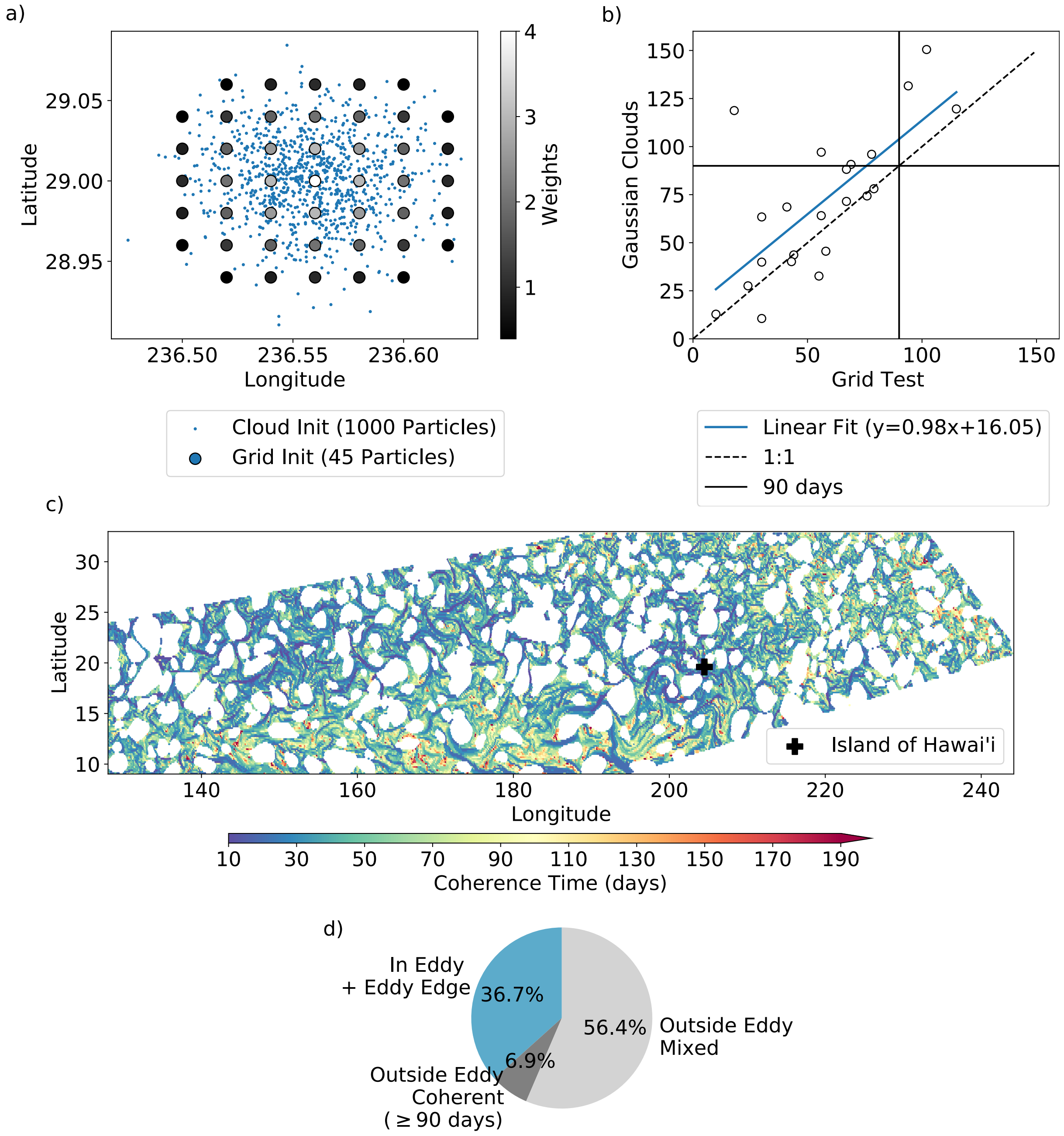

Fig. S11. A gyre-scale calculation of outside-eddy water mass coherence times. **a)** An example of a Gaussian cloud used in the main analysis is shown in blue, with the corresponding 0.02° gridded particle cloud superimposed. The Gaussian cloud is composed of 1,000 particles, while the low-resolution proxy has 45. The low-resolution particles are colored by their weights (ranging from 0.4 - 4) assigned to each particle based on their initialization distance. The weights were used to calculate a weighted mean distance from the center particle for the coherence time calculation, yielding an approximation that mimics the Gaussian cloud estimate but with lower particle resolution. **b)** Comparison of the coherence time calculated from the Gaussian clouds versus the low-resolution method. While they have good agreement (slope = 0.98), the low-resolution method systematically underestimated coherence time, suggesting that the estimated number of coherent water masses (≥ 90 days) across the gyre is conservative. **c)** A map of estimated coherence times of outside-eddy waters across the gyre on November 23, 2021. **d)** The fractions of the NPSG on November 23, 2021 in an eddy or eddy edge (36.7%), in a coherent outside-eddy water mass (6.9%), and in a mixed outside-eddy water mass (56.4%).

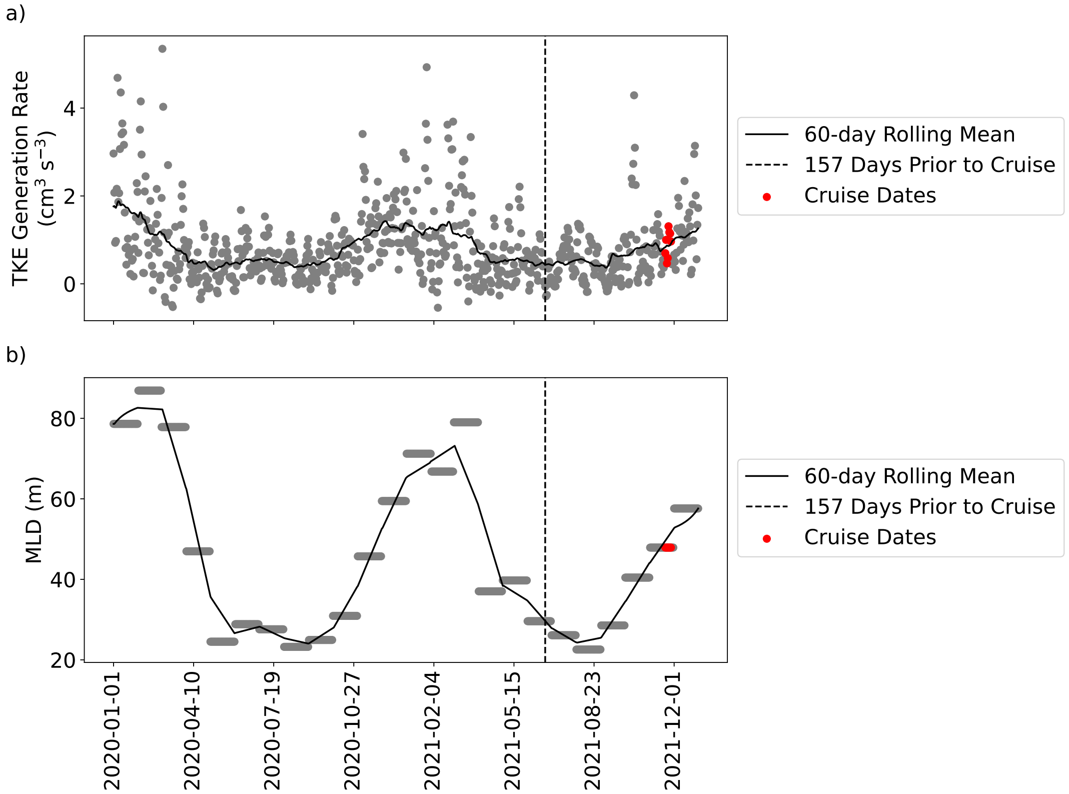

Fig. S12. a) Daily Turbulent Kinetic Energy (TKE) generation rate from January 1, 2020 to December 31, 2021. The scatter points represent a mean within the latitudinal bounds of 15 to 35°N and longitudinal bounds of 215 to 240. The dates of the cruise sampling are highlighted in red. The dashed line aligns with the maximum coherence time (157 days) backward in time from the sampling dates. **b)** The monthly mixed layer depth (MLD).

Table S1. Sample metadata. Samples are labeled with a non-zero identification number if located in an eddy or on an eddy edge.

Sample ID	Date (YYYYMMDD)	Time (UTC)	Latitude (°N)	Longitude	In Anti	Anti Edge	In Cyc	Cyc Edge	Coherence Time (d)
16	20211120	10:54	29.0	236.5574	0	0	0	0	32.67
17	20211120	12:44	28.739	236.1088	0	0	0	0	43.67
18	20211120	14:43	28.503	235.714	0	0	0	0	40.17
19	20211121	02:03	28.2847	235.3517	0	0	0	0	97.08
20	20211121	04:04	28.0397	234.9534	0	0	0	0	63.42
21	20211121	06:05	27.8012	234.5413	0	0	849094	0	50.42
22	20211121	08:00	27.5684	234.1578	0	0	0	849094	49.0
23	20211121	09:53	27.3373	233.7767	0	0	0	0	45.58
24	20211121	11:53	27.0992	233.374	0	0	0	0	12.83
25	20211121	18:33	26.8725	233.0058	0	0	831329	0	79.67
26	20211121	20:35	26.652	232.6401	0	0	831329	0	73.17
27	20211121	22:25	26.4314	232.2805	0	0	831329	0	53.08
28	20211122	00:41	26.1498	231.8127	0	0	0	0	61.17
29	20211122	02:10	25.9711	231.5141	0	0	0	0	39.92
30	20211122	04:03	25.7446	231.1433	0	0	0	0	119.58
31	20211122	05:58	25.516	230.766	0	0	0	0	78.08
32	20211122	07:59	25.282	230.387	0	0	0	0	64.08
33	20211122	09:55	25.058	230.012	0	0	0	0	74.33
34	20211122	11:35	24.837	229.662	0	0	0	0	71.5
35	20211122	13:13	24.6388	229.34	0	0	0	0	90.75
36	20211122	15:27	24.369	228.907	0	0	0	844202	64.75
37	20211122	17:22	24.135	228.525	0	0	844202	0	130.5
38	20211122	19:13	23.906	228.154	0	0	844202	0	70.08
39	20211122	21:12	23.6708	227.7761	0	0	844202	0	156.17
40	20211122	23:16	23.4384	227.3968	0	0	0	0	44.83
41	20211123	01:25	23.1732	226.9761	0	0	0	0	96.0
44	20211123	03:15	22.943	226.61	0	799833	0	0	53.58
45	20211123	05:15	22.701	226.225	799833	0	0	0	62.58
44R	20211123	07:10	22.469	225.848	799833	0	0	0	35.58
46	20211123	09:07	22.232	225.47	799833	0	0	0	17.58
47	20211123	11:09	21.9817	225.0713	799833	0	0	0	22.25
48	20211123	13:02	21.7525	224.7086	0	0	0	0	27.58
49	20211123	20:30	21.523	224.3435	0	0	823188	0	55.92
50	20211123	22:21	21.2948	223.9874	0	0	823188	0	73.67
51	20211124	00:20	21.0504	223.6003	0	0	823188	0	99.58
52	20211124	02:07	20.8288	223.2527	0	0	823188	0	71.33
53	20211124	04:11	20.5925	222.8783	0	0	823188	0	26.33
54	20211124	06:03	20.3584	222.5132	0	0	0	823188	28.08
55	20211124	08:00	20.127	222.1	0	0	0	0	22.25
56	20211124	10:00	19.875	221.754	0	0	847844	0	82.08
57	20211124	21:37	19.6384	221.3856	0	0	847844	0	40.75
58	20211126	01:52	19.3508	221.2797	0	0	0	847844	42.5
59	20211126	04:04	19.025	220.9163	0	0	0	0	88.17
60	20211126	06:00	18.732	220.6	0	0	0	0	131.58
61	20211126	07:58	18.446	220.28	0	0	0	0	10.58
62	20211126	09:40	18.1839	219.9955	0	0	0	0	42.17
64	20211126	11:32	17.9047	219.6855	0	0	845344	0	38.08
66	20211126	18:28	17.6862	219.8291	0	0	845344	0	14.67
67	20211126	20:58	17.282	219.9525	0	0	845344	0	110.17
68	20211126	23:04	16.8736	219.995	0	0	0	845344	154.0
69	20211127	01:02	16.4513	219.9996	0	0	0	0	150.5
70	20211127	03:00	16.036	219.9977	0	0	0	0	118.75
71	20211127	04:58	15.6172	220.0006	0	0	0	0	68.58

Table S2. Eddy attributes.

Name	AVISO ID#	Polarity	Birth Day	Death Day	Lifespan (days)	Crossing Date	Crossing Age
Shrutie	849094	Cyc	11/21/21	12/16/21	26	11/21/21	1
Zinka	831329	Cyc	04/12/21	*	304+	11/21-11/22/21	223-224
Dean	844202	Cyc	09/21/21	*	142+	11/22/21	63
Josh	799833	Anti	11/12/21	01/06/22	56	11/23/21	12
Sharon	823188	Cyc	01/02/21	*	404+	11/23-11/24/21	326-327
Cathy	847844	Cyc	11/05/21	02/07/22	95	11/24-11/26/21	20-22
Koko	845344	Cyc	10/05/21	*	128+	11/26/21	53

* Eddy still existed on the last day of the AVISO dataset (02/09/22).

Table S3. Phytoplankton group statistics (Mean: $\overline{X}$; Standard Deviation: σ; CI: 95% Confidence Interval; units: gene copies/mL). Shading indicates significance where for green the CI is entirely positive, and for purple the CI is entirely negative.

	Abundance $\overline{X}$	Eddy & Edge STA $\overline{X}$	Eddy & Edge STA σ	Outside Mixed STA $\overline{X}$	Outside Mixed STA σ	Outside Coherent STA $\overline{X}$	Outside Coherent STA σ
Cyanobacteria	223682.7	10839.6 CI:[9319.5,12298.0]	21237.4	13610.5 CI:[11575.2,15704.1]	21774.5	345.6 CI:[-3974.1,4585.4]	21318.7
All Eukaryotes	20453.1	813.8 CI:[732.2,881.4]	1753.4	1175.2 CI:[1076.8,1270.1]	1748.6	-2418.3 CI:[-2605.2,-2225.3]	1231.9
Prymnesiophyte	7582.6	416.0 CI:[372.3,452.9]	943.7	548.0 CI:[503.6,591.4]	894.0	-988.2 CI:[-1075.6,-894.4]	724.0
Dinoflagellate	4220.3	80.0 CI:[26.5,129.9]	580.0	252.4 CI:[182.6,324.5]	488.9	-583.9 CI:[-720.2,-445.6]	385.5
Dictyochophyte	3907.0	98.6 CI:[72.8,120.9]	389.7	195.8 CI:[162.4,227.5]	530.6	-433.6 CI:[-499.8,-369.6]	445.1
Chrysophyte	2369.3	137.8 CI:[119.4,155.4]	255.6	105.4 CI:[81.8,127.3]	268.5	-223.5 CI:[-267.8,-178.9]	173.7
Pelagophyte	602.4	34.8 CI:[24.8,43.8]	69.1	18.9 CI:[5.2,33.5]	70.4	-145.9 CI:[-175.7,-114.8]	115.8
Diatom	307.4	20.5 CI:[14.0,26.3]	63.4	-9.8 CI:[-19.6,0.4]	57.9	-0.7 CI:[-22.7,22.5]	57.5
Chlorophyte	207.3	-1.5 CI:[-6.3,3.7]	63.6	8.7 CI:[0.7,16.0]	75.1	7.9 CI:[-5.0,20.7]	62.5
Prasinodermophyte	50.9	-0.5 CI:[-2.8,1.6]	19.9	4.3 CI:[1.4,7.3]	19.8	-1.0 CI:[-6.2,4.3]	19.8
Cryptophyte	2.3	0.2 CI:[-0.1,0.5]	2.3	-0.6 CI:[-1.3,0.1]	5.6	1.8 CI:[0.5,3.1]	5.7
Unknown Eukaryote	1203.6	28.0 CI:[17.7,38.4]	139.4	51.9 CI:[37.5,65.5]	173.5	-51.2 CI:[-77.9,-22.2]	84.0

Table S4. ASV average abundance and significance in three mesoscale niches.

Movie S1. 150-day backward Lagrangian trajectories of the Gaussian clouds initialized at each sample site. Outside-eddy samples have a gray color scheme, cyclones blue, and anticyclones red. Sample cites are labeled with a circle if outside an eddy, a square for inside, and a triangle on eddy edges ($<$ 15km from the boundary). The background is the contours of the mean absolute dynamic topography.

References

1. VW Ekman, On the influence of the Earth's rotation on ocean currents. *Ark. Math. Astron. Phys.* **2** (1905).
2. CMEMS, Global ocean gridded l4 sea surface heights and derived variables reprocessed 1993 ongoing, marine data store (2020).
3. ESR, K Dohan, Ocean surface current analyses real-time (oscar) surface currents - final 0.25 degree (version 2.0). ver. 2.0. (2022).
4. X Liang, M Spall, C Wunsch, Global Ocean Vertical Velocity From a Dynamically Consistent Ocean State Estimate. *J. Geophys. Res. Ocean.* **122**, 8208–8224 (2017).
5. JR Ledwell, AJ Watson, CS Law, Mixing of a tracer in the pycnocline. *J. Geophys. Res. Ocean.* **103**, 21499–21529 (1998).
6. A Okubo, Oceanic diffusion diagrams. *Deep. Res.* **18**, 789–802 (1971).
7. P Niiler, E Kraus, One-dimensional models of the upper ocean in *Modelling and prediction of the upper layers of the ocean*, ed. E Kraus. (Pergamon Press), pp. 143–172 (1977).
8. EB Kraus, JS Turner, A one-dimensional model of the seasonal thermocline II. The general theory and its consequences. *Tellus* **19**, 98–106 (1967).
9. M Follows, S Dutkiewicz, Meteorological modulation of the North Atlantic spring bloom. *Deep. Res. II* **49**, 321–344 (2002).
10. C3S, Era5 hourly data on single levels from 1940 to present (2023).
11. C3S, Oras5 global ocean reanalysis monthly data from 1958 to present (2021).
12. G Haller, A Hadjighasem, M Farazmand, F Huhn, Defining coherent vortices objectively from the vorticity. *J. Fluid Mech.* **795**, 136–173 (2016).
